# A Marine *Brevibacillus*-Derived Membrane-Lytic Peptide: Molecular Insight and Biophysical Characterization of a Novel AMP, FNL62-AMP

**DOI:** 10.3390/md24090309

**Published:** 2026-09-04

**Authors:** Namfa Sermkaew, Apichart Atipairin, Sucheewin Krobthong, Chanat Aonbangkhen, Yodying Yingchutrakul, Jumpei Uchiyama, Nuttapon Songnaka

**Affiliations:** 1 School of Pharmacy, Walailak University, Thasala, Nakhon Si Thammarat 80160, Thailand; namfa.se@wu.ac.th (N.S.); apichart.at@wu.ac.th (A.A.); 2 Drug and Cosmetics Excellence Center, Walailak University, Thasala, Nakhon Si Thammarat 80160, Thailand; 3 Center of Excellence in Natural Products Chemistry (CENP), Department of Chemistry, Faculty of Science, Chulalongkorn University, Pathumwan, Bangkok 10330, Thailand; sucheewin.k@chula.ac.th (S.K.); chanat.a@chula.ac.th (C.A.); 4 Center of Excellence on Petrochemical and Materials Technology, Chulalongkorn University, Pathumwan, Bangkok 10330, Thailand; 5 National Center for Genetic Engineering and Biotechnology, National Science and Technology Development Agency, Khlong Luang, Pathum Thani 12120, Thailand; yodying.yin@biotec.or.th; 6 Department of Infectious Diseases, Graduate School of Medicine, Dentistry and Pharmaceutical Sciences, Okayama University, Okayama 700-8558, Japan; uchiyama@okayama-u.ac.jp

**Keywords:** AMP, antimicrobial peptide, antimicrobial resistance, *Brevibacillus*, FNL62, molecular dynamics simulations, MRSA, *Staphylococcus aureus*, self-aggregation, genome

## Abstract

This study characterizes the genomic identity, functional efficacy, and computational biophysics of FNL62-AMP, a novel antimicrobial peptide isolated from a phylogenomically distinct, newly identified *Brevibacillus* species. Production kinetics revealed a late-exponential phase onset of antibacterial activity with sustained potency against methicillin-resistant *Staphylococcus aureus* (MRSA). LC-MS/MS analysis identified the peptide sequence as NH_2_-LLLLFR-COOH. FNL62-AMP demonstrated excellent formulation resilience, retaining full anti-MRSA activity under high thermal stress (80 °C for 6 h) and showing robust resistance to generic trypsin and proteinase K proteolysis. Formulative co-incubation assays demonstrated charge-dependent compatibility, where nonionic Triton X-100 preserved baseline efficacy while ionic surfactants induced antagonism. In vitro time-kill kinetics, scanning electron microscopy, and SYTOX Green assays confirmed rapid, concentration-dependent bactericidal action driven by immediate membrane permeabilization. Molecular dynamics simulations successfully captured the spontaneous self-assembly of 64 FNL62-AMP monomers into a stable macro-aggregate. This consolidation process was quantitatively characterized by a simultaneous contraction in the radius of gyration (*R_g_*), a sharp drop in solvent-accessible surface area (SASA), and a transitional plateau in mean squared displacement (MSD). Ultimately, the high thermal stability, structural resilience, and predictable surfactant compatibility of FNL62-AMP propose ways to be developed for lead optimization and druggability.

## 1. Introduction

The emergence and rapid dissemination of antimicrobial resistance (AMR) has become a major global health concern, significantly reducing the effectiveness of commonly used antibiotics. This phenomenon contributes to prolonged infections, higher mortality rates, and increased healthcare expenditures. The World Health Organization recognizes AMR as a serious threat to both public health and socio-economic development. In 2019, bacterial AMR was linked to approximately 4.95 million deaths worldwide, with 1.27 million deaths directly caused by resistant infections [1].

The ability of bacteria to rapidly adapt and evade traditional therapies has intensified the need for novel treatment approaches, particularly as multidrug-resistant organisms become prevalent in both hospital and community environments. Notably, the ESKAPE pathogens (*Enterococcus faecium*, *Staphylococcus aureus*, *Klebsiella pneumoniae*, *Acinetobacter baumannii*, *Pseudomonas aeruginosa*, and *Enterobacter* species) demonstrate remarkable resistance to conventional therapies [2]. Among these, methicillin-resistant *S. aureus* (MRSA) poses a particularly serious threat, responsible for severe infections with high mortality [3].

The rapid escalation of global antibiotic resistance necessitates the development of novel antimicrobial agents with distinct mechanisms of action. Unlike traditional antibiotics, antimicrobial peptides (AMPs) have been recognized as promising candidates due to their ability to target multiple low-affinity sites—primarily the bacterial membrane—which helps limit the development of resistance. Most AMPs exert a direct microbicidal effect either by disrupting the integrity of microbial membranes or by translocating across the membrane to interact with intracellular targets [4,5]. The activity of these typically cationic AMPs is largely driven by electrostatic interactions with negatively charged components of microbial membranes, granting them distinct selectivity for bacterial surfaces over host cells [6].

The marine environment represents a vast reservoir for therapeutic discovery. To survive extreme microbial densities (up to 10^6^ CFU/mL), marine organisms have evolved uniquely adapted AMPs as a primary innate defense. Because these marine-derived molecules frequently exhibit structural characteristics distinct from their terrestrial analogs, they offer novel templates for pharmaceutical development against increasingly resistant pathogens [7].

In the search for such novel bioactive agents, the genus *Brevibacillus* has emerged as a particularly prolific candidate. This genus thrives across diverse ecological niches, ranging from terrestrial soils to marine sediments, by utilizing a highly adaptable metabolic framework. The biosynthetic versatility of *Brevibacillus* is well-documented, with the strain yielding potent anti-MRSA peptides [8], defensin-like bacteriocin [9], AMPs that are active against Gram-positive and Gram-negative bacteria [10], and a novel lanthipeptide with broad-spectrum antibacterial, antifungal, and antiviral activity [11]. Consequently, exploring the specialized metabolites of marine-derived *Brevibacillus* isolates presents a strategic opportunity to identify compounds with unique functional profiles.

The present study reports the comprehensive characterization of this novel cationic peptide, designated FNL62-AMP, demonstrating its potent activity against MRSA. The physicochemical and structural properties of FNL62-AMP were evaluated in detail alongside genome-based analyses of the FNL62 isolate to identify its associated biosynthetic gene clusters. Furthermore, molecular dynamics simulations were employed to elucidate the atomic-level interactions between the peptide and the bacterial membrane. Ultimately, these combined in vitro and in silico findings highlight the potential of FNL62-AMP as a promising early-stage lead peptide, paving the way for the development of novel antimicrobial agents targeting drug-resistant staphylococcal infections.

## 2. Results

### 2.1. Growth Kinetics and Time-Dependent Antimicrobial Activity of the FNL62 Isolate Crude Supernatant

The FNL62 strain was isolated as a single colony from mangrove sediment following cultivation on Mueller–Hinton (MH) agar at 30 °C for 24 h. Preliminary screening of the 24 h cell-free supernatant (CFS) via an agar well diffusion assay demonstrated antibacterial activity against *Staphylococcus aureus* TISTR 517 and three methicillin-resistant *S. aureus* (MRSA) strains (142, 1096, and 2468). To define the antibacterial spectrum and determine the optimal culture duration for maximum active compound yield, the growth kinetics of the isolate were correlated with its antibacterial efficacy using an activity-based assay. The relationship between the growth kinetics of the FNL62 isolate (optical density at 625 nm; OD_625_) and the time-dependent production of its antibacterial compounds was monitored over a 168 h incubation period against a panel of Gram-positive and Gram-negative indicator strains.

**Figure 1:**
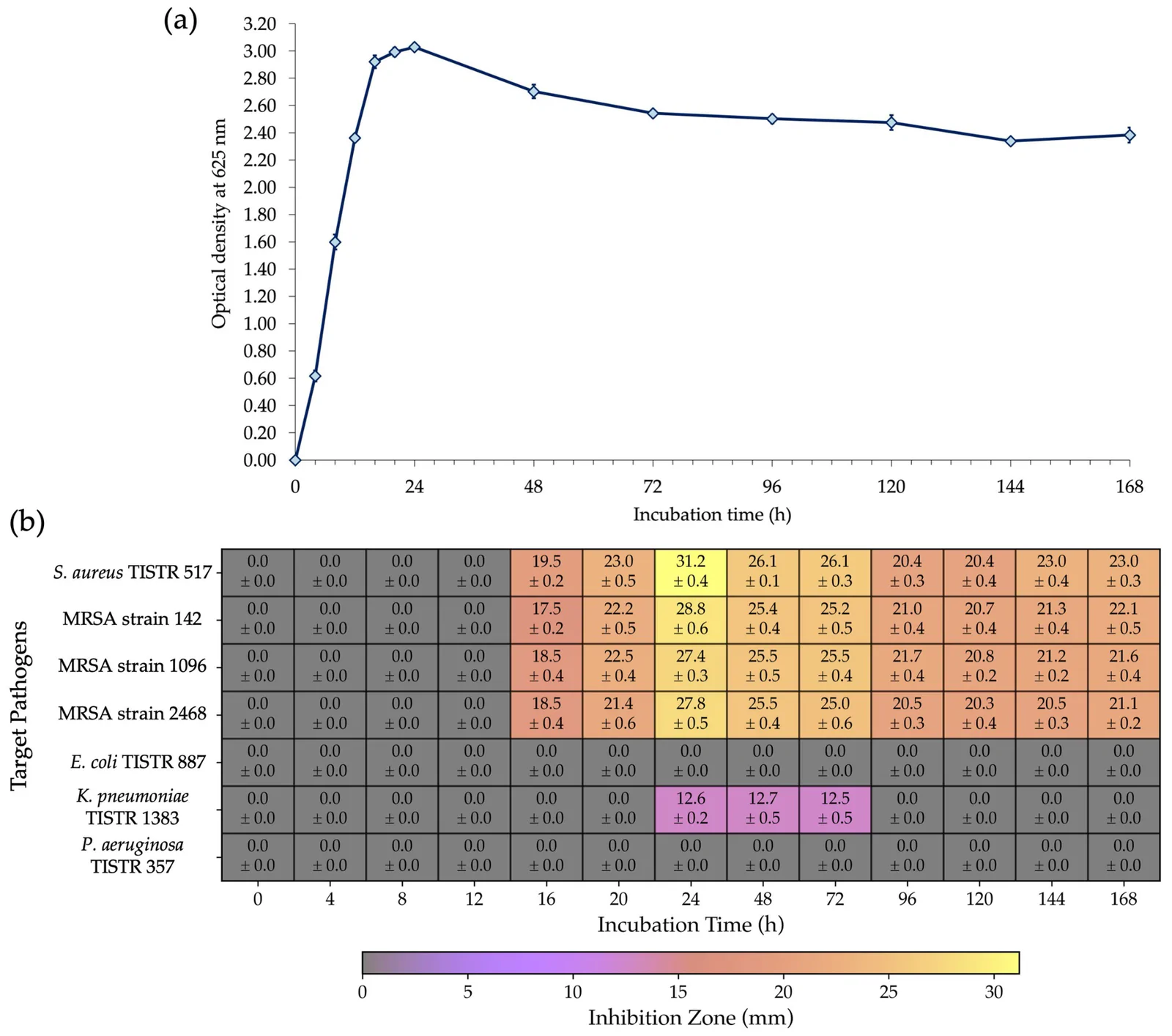
Growth kinetics of the FNL62 isolate correlated with the time-dependent antimicrobial activity of its crude supernatant over 168 h. The primary axis depicts the optical density (OD_625_) of the FNL62 isolate culture across different growth phases (**a**). The heatmap illustrates the antimicrobial activity, measured as the inhibition zone diameter (mm), against a panel of Gram-positive indicators (*S. aureus* TISTR 517 and MRSA strains 142, 1096, and 2468) and Gram-negative indicators (*K. pneumoniae* TISTR 1383, *P. aeruginosa* TISTR 357, and *E. coli* TISTR 887) (**b**). Data points represent the mean ± standard deviation (SD) (*n* = 3).

The OD_625_ of the FNL62 isolate culture increased exponentially from 0 to 16 h, corresponding to a logarithmic growth phase. Following this exponential phase, the growth curve transitioned toward a stationary phase plateau, with the OD_625_ reaching its maximum at 24 h. From 24 to 72 h, a slight decline in optical density was observed, after which the OD_625_ remained relatively constant through 168 h (Figure 1a). The onset of antimicrobial compound production correlated closely with the late exponential phase. Initial antibacterial activity against *S. aureus* TISTR 517 and the three MRSA strains (142, 1096, and 2468) was detected between 16 and 20 h of incubation. Statistical analysis involving multiple comparisons (*p* < 0.05) across incubation periods for each tested pathogen confirmed that the maximum zones of inhibition against all Gram-positive indicators were observed at 24 h, coinciding with the early stationary phase of bacterial growth. Beyond this 24 h optimum, anti-MRSA activity gradually declined, demonstrating a stable but lower baseline of inhibitory efficacy between 96 and 168 h.

Regarding the spectrum of action against Gram-negative indicators, antibacterial efficacy was strictly limited to *Klebsiella pneumoniae* TISTR 1383, with no inhibitory activity detected against *Pseudomonas aeruginosa* TISTR 357 or *Escherichia coli* TISTR 887 at any evaluated time point. Notably, this anti-*K. pneumoniae* activity was highly time-restricted, appearing exclusively during the 24- to 72 h window (Figure 1b). The kinetic profiles demonstrate that the FNL62 isolate has potential for the antibacterial compounds utilization with pronounced activity against Gram-positive pathogens, including *S. aureus* and antibiotic-resistant strains such as MRSA.

### 2.2. Purification of the Antibacterial Components of FNL62 Isolate and Structural Analysis of the Bioactive Peptide

**Figure 2:**
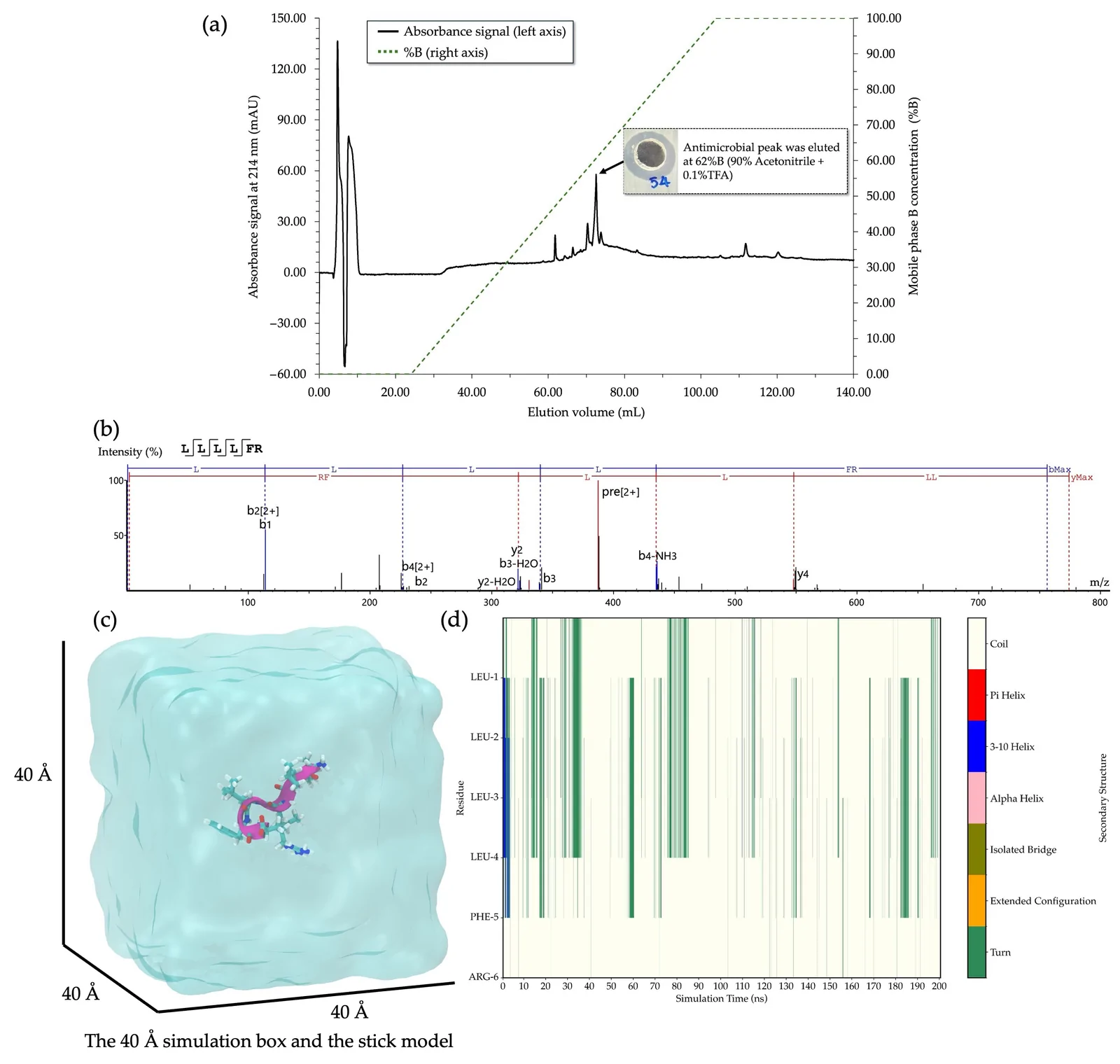
Isolation, structural identification, and conformational dynamics of bioactive FNL62-AMP. Reversed-phase chromatographic (RPC) purification profile of FNL62-AMP. The chromatogram displays the relationship between the elution volume (*x*-axis), the ultraviolet (UV) absorbance signal monitored at 214 nm indicating peptide bonds (left *y*-axis), and the linear gradient concentration of mobile phase B (90% acetonitrile in 0.1% TFA; right *y*-axis). The arrow denotes the active fraction pooling window eluting at 62% mobile phase B, where anti-MRSA activity was successfully localized and recovered (**a**). LC-MS/MS mass spectrum and sequence identification of the purified peptide. The tandem mass spectrometry (MS/MS) fragmentation pattern defines the amino acid sequence of the isolated bioactive peptide, with labeled fragment ions (*b* and *y* series) confirming the primary structural assignment (**b**). Three-dimensional molecular snapshot of free FNL62-AMP from MD simulations in an explicit aqueous solvent in a 40 × 40 × 40 Å simulation box; the atomistic model, represented using a CPK coloring style (**c**). Time-resolved per-residue secondary structure evolution of FNL62-AMP in an aqueous environment. The *x*-axis represents the total molecular dynamics simulation time (ns), the primary *y*-axis (left) indexes individual amino acid residues from the N- to C-terminus, and the secondary *y*-axis details the specific secondary structure conformations (including coil, π-helix, 3_10_-helix, α-helix, isolated bridge, extended configuration, and turn) color-coded across the trajectory (**d**).

An orthogonal purification strategy was applied to 987.54 mL of the crude CFS from the 24 h FNL62 culture. Following protein precipitation via ammonium sulfate, the 50% saturation fraction exhibited the highest antibacterial activity against MRSA strain 2468. This active precipitate was redissolved, dialyzed, and subjected to further purification using cation-exchange chromatography. During cation-exchange chromatography, the active fraction eluted as a distinct peak at a retention time of 25.98 min under isocratic conditions. This active peak was collected and subjected to a final purification step via reversed-phase chromatography (RPC), where the target compound eluted at 62% mobile phase B (at an elution volume of 72.56 mL) (Figure 2a). Antibacterial screening of the collected fractions against MRSA strain 2468 confirmed that the activity was recovered within this specific chromatographic peak. The sequential purification process successfully isolated the major antimicrobial component, achieving a final yield of 11.84% relative to the initial batch culture. Overall, the procedure achieved a 12.44-fold purification compared to the crude cell-free supernatant (CFS) of the 24 h FNL62 culture, ultimately yielding 9.18 mg of the purified antimicrobial peptide from 987.54 mL of starting CFS (Table 1).

Subsequent LC-MS/MS analysis identified the amino acid sequence of the bioactive peptide as NH_2_-LLLLFR-COOH (designated FNL62-AMP), with an experimentally determined molecular weight of 774.02 Da and an average local alignment confidence score of 78.5% (Figure 2b, Tables S1 and S2). Physicochemical predictions using the APD database indicated that the peptide is amphipathic, possessing a net positive charge of +1 and a high hydrophobic residue content of 83%. A sequence homology search within the APD database revealed a 50% similarity to Gageotetrin B (isolated from *Bacillus subtilis*) and the de novo designed peptide L4K2W^4^. Notably, searches against the DRAMP and DBAASP databases yielded no sequence matches, underscoring the potential novelty of the FNL62-AMP sequence [12,13]. The conformational dynamics of FNL62-AMP were investigated using molecular dynamics (MD) simulations in an explicit aqueous solvent. The evolution of the peptide’s secondary structure was monitored every nanosecond (ns) over a 200 ns trajectory. Representative three-dimensional snapshots illustrate FNL62-AMP in a coil conformation, floating freely in bulk water containing 0.15 M NaCl (Figure 2c). To quantify the structural changes over time, a time-resolved conformational profile for each amino acid residue was mapped across the entire 200 ns simulation (Figure 2d). The trajectory revealed that the peptide remains fundamentally disordered in aqueous solution. Throughout the simulation, FNL62-AMP predominantly adopted random coil and transient turn configurations without transitioning into more ordered secondary structures.

### 2.3. Minimum Inhibitory and Bactericidal Concentrations, and Time-Kill Kinetics of FNL62-AMP Against S. aureus and MRSA

In the broth microdilution assay, FNL62-AMP exhibited a minimum inhibitory concentration (MIC) of 0.5 µg/mL against *S. aureus* TISTR 517, whereas a two-fold higher concentration (1.0 µg/mL) was required to inhibit the MRSA strains. The minimum bactericidal concentration (MBC) of the peptide was determined to be four-fold higher than its respective MICs across all tested strains. The growth-inhibitory activity of FNL62-AMP was comparable to that of the standard antibiotic vancomycin; however, vancomycin achieved complete bacterial eradication (MBC) at the same concentration as its MIC. As expected, the resistance phenotype of the MRSA strains was confirmed using cefoxitin, which successfully inhibited the susceptible *S. aureus* TISTR 517 strain but failed to suppress the resistant variants (Table 2).

**Figure 3:**
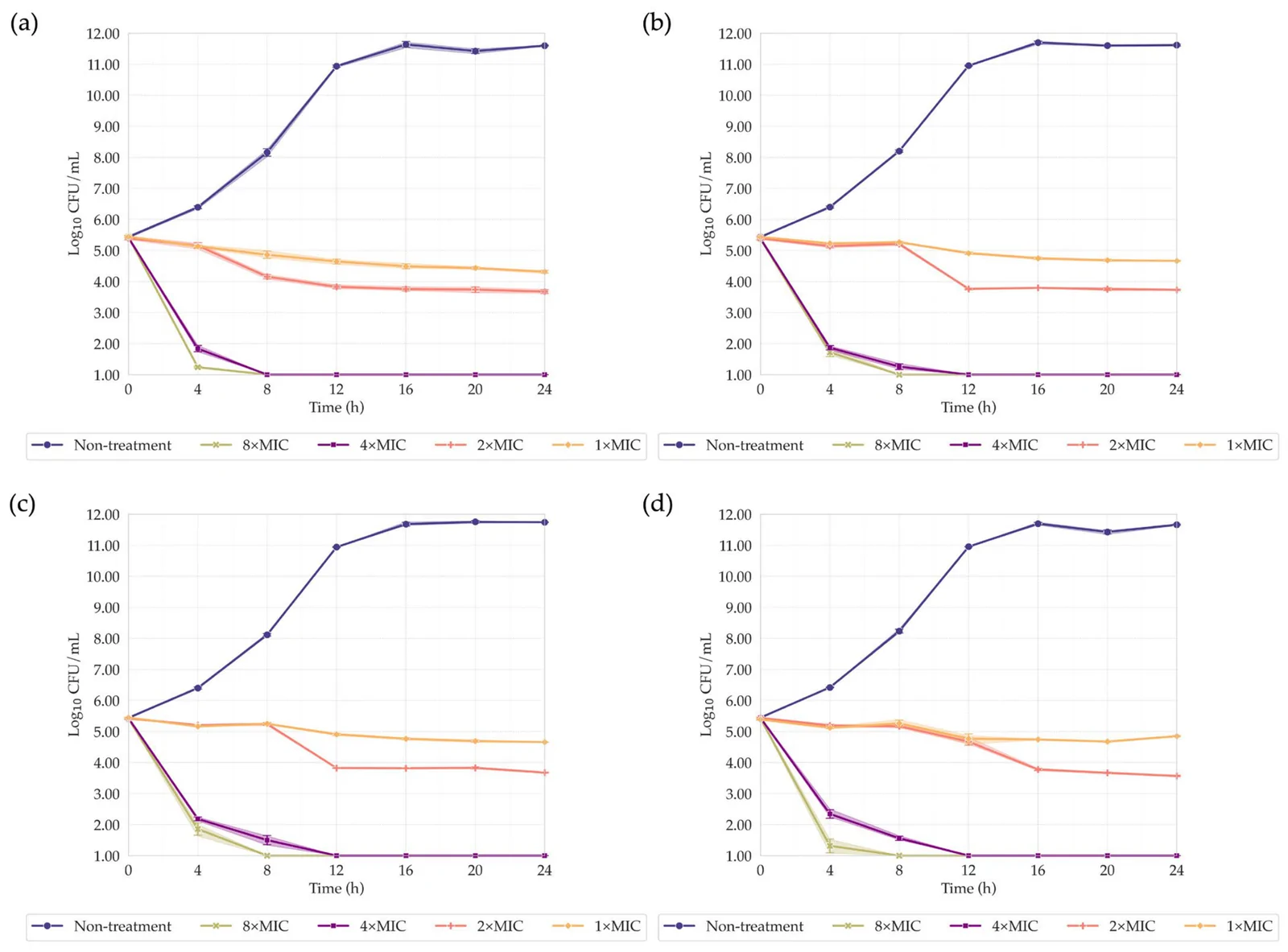
Time-kill kinetics of FNL62-AMP against susceptible and resistant *S. aureus* strains. Cell viability of *S. aureus* TISTR 517 (**a**), MRSA strain 142 (**b**), MRSA strain 1096 (**c**), and MRSA strain 2468 (**d**) was enumerated over a 24 h FNL62-AMP exposure period. Surviving cells are expressed as log_10_ CFU/mL following treatment with FNL62-AMP at 1× MIC (◆), 2× MIC (+), 4× MIC (■), and 8× MIC (×), compared to an untreated control (●). Data points represent the mean ± SD from three independent experiments (*n* = 3), and shaded areas indicate the 95% confidence intervals. The limit of detection (LOD) was 1 log_10_CFU/mL, corresponding to the minimum value on the *y*-axis.

The time-kill kinetics of FNL62-AMP were evaluated against *S. aureus* TISTR 517 and three MRSA strains (142, 1096, and 2468) over a 24 h incubation period with a limit of detection of 1 log_10_CFU/mL (Figure 3). Overall, the peptide exhibited a concentration-dependent mode of action, markedly reducing bacterial viability compared to the typical exponential growth observed in the untreated control. At the highest evaluated concentration (8× MIC), a rapid loss of viability was observed across all tested strains, culminating in complete bacterial eradication within 8 h of treatment. During this rapid bactericidal phase, the calculated bacterial reduction rates for *S. aureus* TISTR 517, MRSA 142, MRSA 1096, and MRSA 2468 were 0.5546 ± 0.1080, 0.5463 ± 0.0803, 0.5513 ± 0.0744, and 0.5521 ± 0.1041 log_10_ CFU/h, respectively. A similar bactericidal endpoint was achieved at 4× MIC, where complete bacterial eradication across all four strains was shifted to the 12 h time point. The corresponding bacterial reduction rates at this concentration were 0.5521 ± 0.0750, 0.3440 ± 0.0612, 0.3493 ± 0.0523, and 0.3526 ± 0.0480 log_10_ CFU/h for *S. aureus* TISTR 517, MRSA 142, MRSA 1096, and MRSA 2468, respectively. In contrast, treatment at lower concentrations (1× and 2× MIC) was bacteriostatic rather than completely bactericidal, failing to achieve total microbial clearance within the 24 h window. However, the 2× MIC treatment demonstrated a progressively faster reduction rate and a greater overall decrease in viability than the 1× MIC group. Specifically, the reduction rates at 2× MIC were 0.0749 ± 0.0088, 0.0819 ± 0.0099, 0.0841 ± 0.0095, and 0.0897 ± 0.0062 log_10_ CFU/h for *S. aureus* TISTR 517, MRSA 142, MRSA 1096, and MRSA 2468, respectively. At 1× MIC, the reduction rates were visibly slower, recorded at 0.0460 ± 0.0031, 0.0350 ± 0.0024, 0.0338 ± 0.0024, and 0.0270 ± 0.0045 log_10_ CFU/h, respectively. For both low-dose treatment groups, bacterial viability plateaued and remained relatively stable after 12 h of incubation.

### 2.4. Evaluation of Membrane Permeability Alterations in S. aureus and MRSA Induced by FNL62-AMP Using the SYTOX Green Uptake Assay

**Figure 4:**
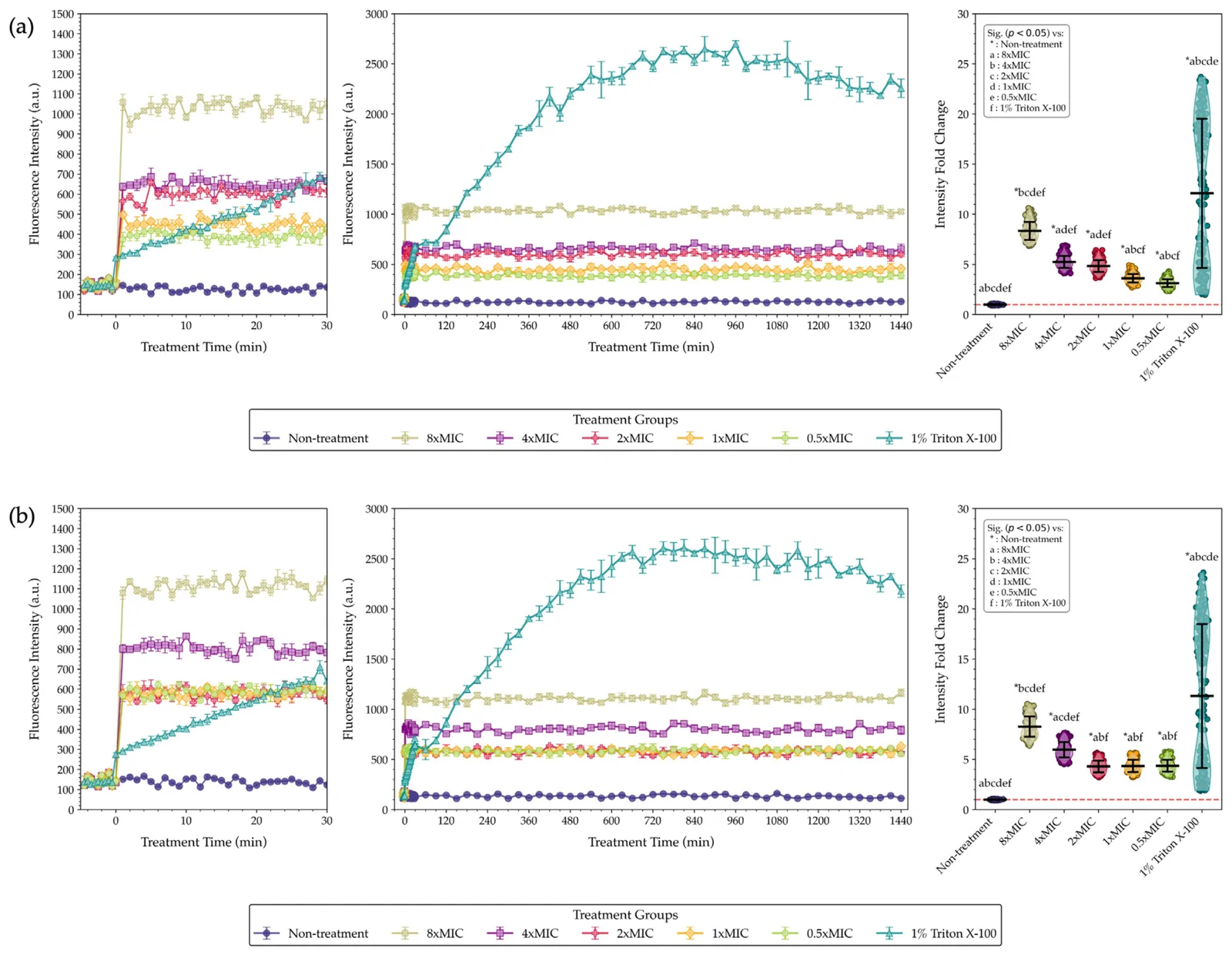
Membrane permeabilization kinetics of FNL62-AMP against *S. aureus* TISTR 517 and MRSA strain 2468. SYTOX Green fluorescence was used to monitor membrane integrity in *S. aureus* TISTR 517 (**a**) and MRSA strain 2468 (**b**) following exposure to FNL62-AMP at concentrations ranging from 0.5× to 8× MIC. For each strain, the left panels provide an expanded view of the initial 30 min to illustrate the rapid onset of membrane disruption; the middle panels display the full 24 h kinetic profiles. The right panels express permeabilization as the fold change in fluorescence intensity relative to the untreated control, using violin plots to visualize the data distribution density and the mean ± SD derived from the pooled time-point readings recorded across the 24 h assay period.

Membrane integrity alterations in *S. aureus* TISTR 517 and MRSA strain 2468 were monitored via a SYTOX Green uptake assay over a 24 h period (Figure 4). Prior to peptide treatment, baseline fluorescence was recorded for 5 min to confirm initial membrane integrity. Following treatment, a distinct concentration-dependent increase in fluorescence intensity was observed across the evaluated AMP concentrations (0.5×, 1×, 2×, 4×, and 8× MIC), with all treatment groups exhibiting significant membrane permeation compared to the untreated control. Exposure to the AMP induced a rapid increase in fluorescence, which subsequently reached a stable plateau (Figure 4a and Figure 4b, left and middle panels, respectively).

Overall, the membrane permeabilization kinetics induced by FNL62-AMP were comparable between *S. aureus* TISTR 517 and MRSA strain 2468. At the highest concentration (8× MIC), both strains exhibited a matching eight-fold increase in fluorescence intensity relative to the untreated control. Similarly, the degree of permeabilization remained comparable between both strains at lower concentrations (0.5× to 4× MIC). However, statistical analysis revealed differing concentration-dependent trends between the two pathogens. In *S. aureus* TISTR 517, the fluorescence fold changes were significantly different across all individual concentrations from 0.5× to 8× MIC. Conversely, in MRSA strain 2468, the fold changes observed at the lower concentration range (0.5× to 2× MIC) did not show statistically significant differences (Figure 4a,b, right panels). Furthermore, comparing FNL62-AMP kinetics to the positive control (1% Triton X-100) revealed distinct mechanisms of membrane disruption. FNL62-AMP induced a rapid initial influx of SYTOX Green that quickly plateaued in a concentration-dependent manner, indicative of the immediate formation of localized membrane defects or pores. In contrast, the surfactant Triton X-100 exhibited a slower initial onset followed by a massive, unconstrained increase in fluorescence during the late treatment phase, characteristic of continuous membrane micellization and ultimate structural dissolution. This distinct kinetic profile could be proposed that FNL62-AMP destabilizes the bilayer without completely solubilizing it.

### 2.5. Ultrastructural Morphological Alterations of S. aureus and MRSA Induced by FNL62-AMP

**Figure 5:**
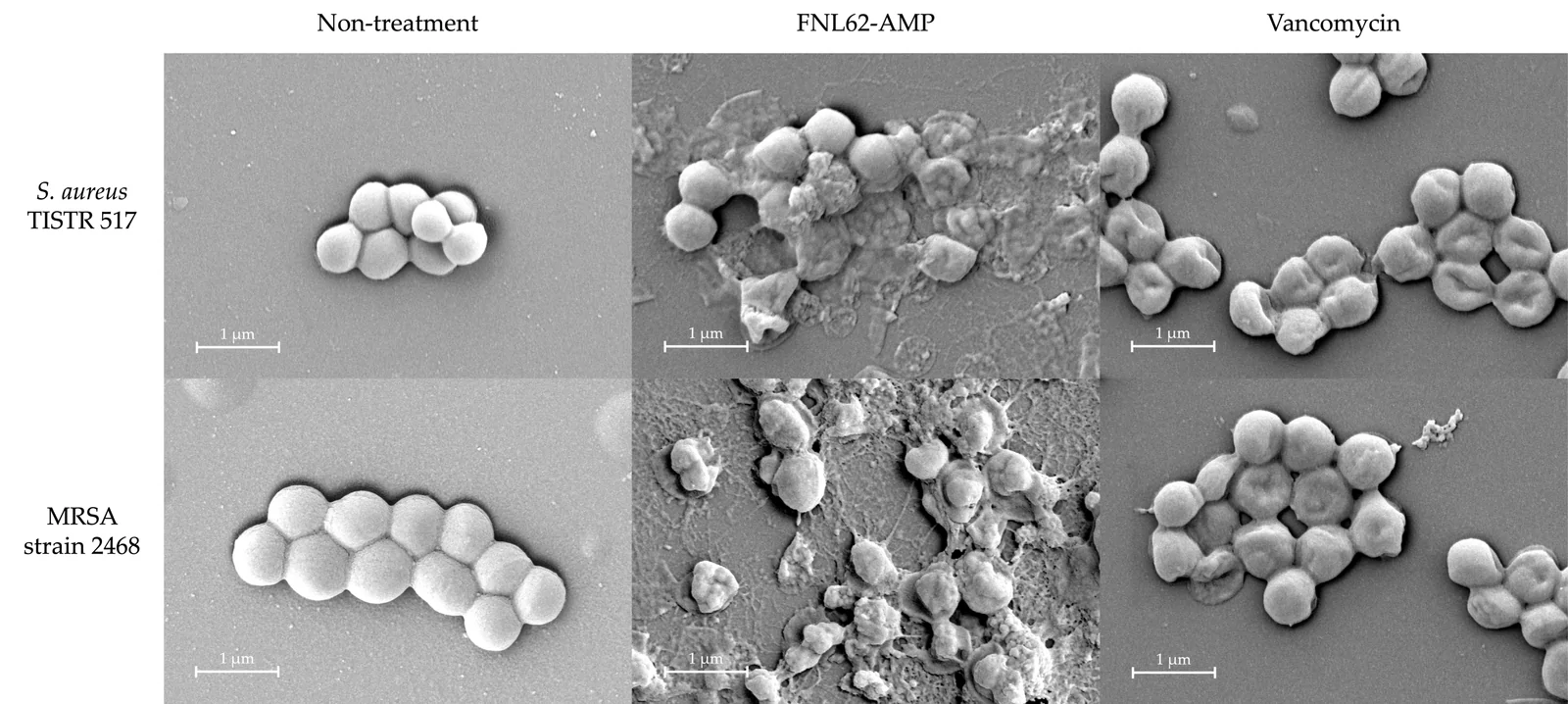
Scanning electron micrographs showing the ultrastructural alterations of *S. aureus* TISTR 517 and MRSA strain 2468. The imaging panel includes untreated controls for both strains, treatment groups exposed to FNL62-AMP (1× MIC for 16 h), and reference groups treated with the standard antibiotic vancomycin (1× MIC for 16 h). All images were acquired at 20,000× magnification (scale bar = 1 µm).

Scanning electron microscopy (SEM) was employed to evaluate the ultrastructural morphological alterations induced in *S. aureus* TISTR 517 and MRSA strain 2468 following treatment with FNL62-AMP or vancomycin, both at a concentration of 1× MIC (Figure 5). The untreated control cells for both strains exhibited characteristic intact, spherical structures with highly uniform, smooth outer surfaces. In contrast, treatment with the AMP induced profound structural damage, characterized by widespread cell lysis, severely shrunken or collapsed cellular architecture, and extensively corrugated surfaces. These morphological changes strongly indicate a direct membrane-disrupting mechanism underlying the peptide’s bactericidal activity. Cells treated with the reference standard, vancomycin, presented a distinctly different morphological profile. Although these cells appeared significantly shrunken and exhibited prominent surface depressions, their outer surfaces remained noticeably smoother and more cohesive than those subjected to the AMP.

### 2.6. Stability Profiling of FNL62-AMP

The stability and robust performance of FNL62-AMP were evaluated by measuring its residual antibacterial activity against MRSA strain 2468 (at a final concentration of 64 µg/mL) following exposure to diverse environmental stressors including thermal stress, enzymatic digestion, surfactant interactions, and pH variations over a time course of 1, 6, and 12 h (Table 3).

FNL62-AMP demonstrated pronounced thermal stability. Incubation at moderate temperatures (40 °C and 60 °C) for up to 12 h resulted in no significant loss of anti-MRSA activity compared to the untreated control maintained at 25 °C. At an elevated temperature of 80 °C, the peptide remained stable at 1 and 6 h, exhibiting a significant decrease in potency only after 12 h of incubation. Exposure to 100 °C caused an immediate reduction in activity at 1 and 6 h, with the lowest residual activity recorded at the 12 h mark across all standard thermal conditions. Under extreme autoclaving conditions (121 °C at 15 psi), the antibacterial efficacy of FNL62-AMP was significantly compromised at all evaluated time points (15 min, 30 min, and 1 h), with the 1 h incubation group displaying a statistically greater loss of activity than the 15 min group.

The susceptibility of FNL62-AMP to enzymatic degradation was assessed using proteinase K, trypsin, and α-chymotrypsin. FNL62-AMP exhibited remarkable resilience to proteinase K, retaining its full anti-MRSA activity throughout the initial 6 h incubation period. However, a significant decrease in activity was observed when the incubation reached 12 h. Conversely, trypsin-treated FNL62-AMP displayed robust tolerance, maintaining stable activity at 12 h compared to the control. The peptide proved vulnerable to α-chymotrypsin cleavage starting at 1 h, and prolonged incubation for 6 and 12 h resulted in a time-dependent decline in residual antimicrobial potency.

Co-incubation with structurally diverse surfactants revealed distinct interaction profiles. To accurately assess these interactions, the intrinsic antibacterial activity of the 1% surfactant solutions alone was first evaluated and normalized against the untreated FNL62-AMP control (set at 100%). Triton X-100 (nonionic) exhibited no inherent antibacterial efficacy (0.0% relative activity). Conversely, the ionic surfactants SDS (anionic) and CTAB (cationic) demonstrated potent intrinsic bactericidal effects on their own, generating inhibition zones comparable to the untreated peptide. When formulated as a mixture, FNL62-AMP maintained excellent compatibility with Triton X-100, retaining full residual activity comparable to the untreated control at every time point. In contrast, the ionic surfactants negatively impacted peptide performance. The addition of either SDS or CTAB to the peptide induced a rapid reduction in the overall anti-MRSA activity of the mixture within 1 h, with a continued decline observed up to 12 h. Notably, the most severe overall loss of activity occurred in the presence of CTAB.

Regarding pH variations, FNL62-AMP exhibited optimal stability under near-neutral conditions, maintaining full efficacy between pH 6.0 and 7.4 for up to 12 h. In acidic environments (pH 1.0, 2.0, and 4.0), a clear trend was observed where residual activity decreased progressively with descending pH values. Under alkaline conditions (pH 10.0 to 12.0), a significant reduction in anti-MRSA activity was detected only after 12 h of exposure. Under extreme basic stress (pH 14.0), FNL62-AMP resisted degradation for up to 1 h, after which its antimicrobial potency gradually declined.

### 2.7. Genomic Characterization and Biosynthetic Potential of Brevibacillus sp. FNL62

**Figure 6:**
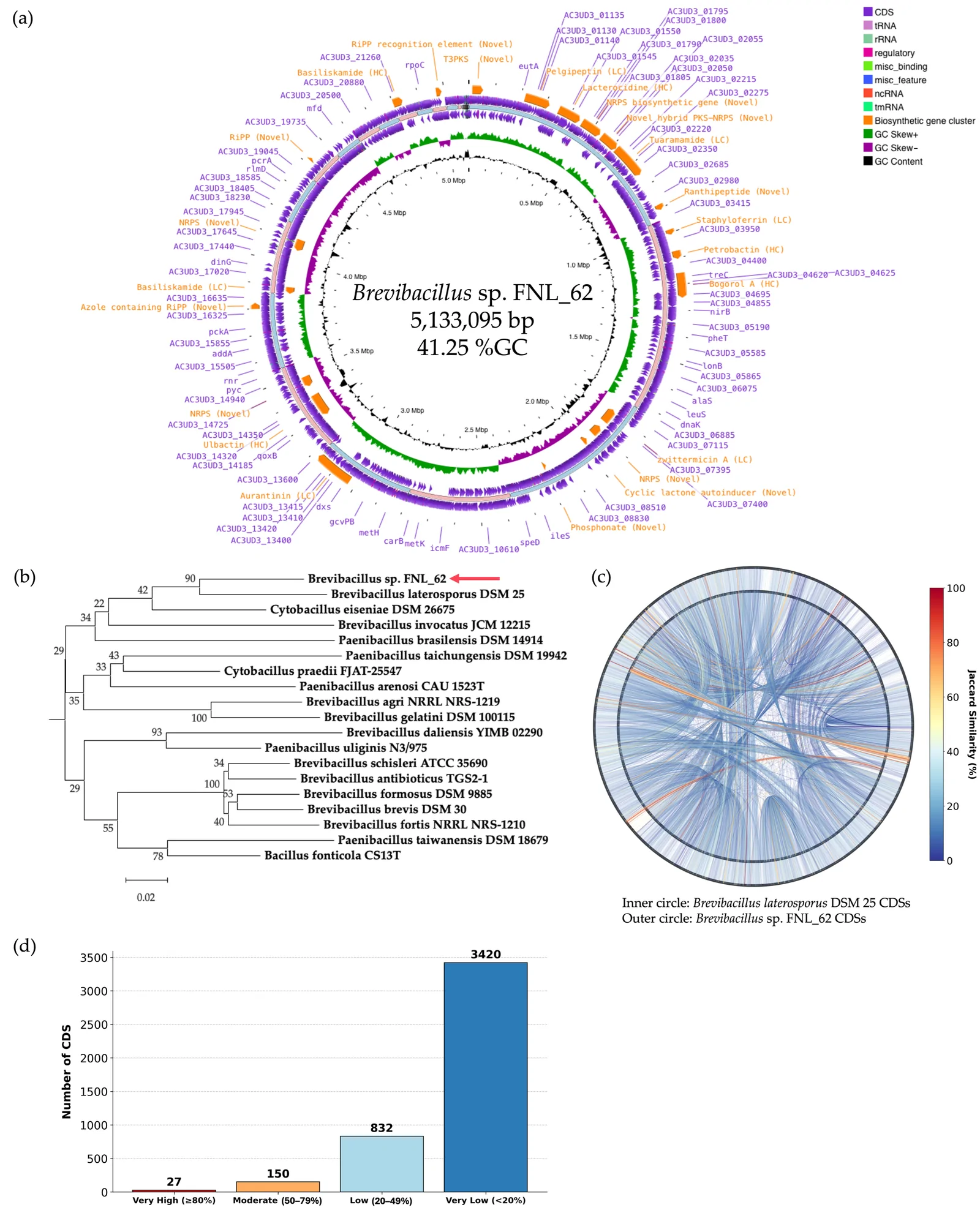
Genomic and phylogenomic analysis of *Brevibacillus* sp. FNL_62. Circular genome map of *Brevibacillus* sp. FNL_62, highlighting key genomic features and biosynthetic gene clusters (BGCs) for antimicrobial compounds and secondary metabolites (**a**). Phylogenomic tree showing the evolutionary relationship of strain FNL62 within the genus *Brevibacillus* (**b**). Circular synteny map representing homologous CDS matching between the *Brevibacillus* sp. FNL_62 genome (outer circle) and *Brevibacillus laterosporus* DSM 25 (inner circle) (**c**). Distribution of the matched CDSs, categorized into four distinct levels of sequence similarity (**d**).

Morphological analysis of a single colony revealed that the FNL62 isolate is a Gram-positive, endospore-forming bacillus. Subsequent genotypic characterization was performed via whole-genome analysis. The genomic assembly of strain FNL62 yielded a total sequence length of 5,133,095 bp distributed across 108 contigs, with a GC content of 41.25%, 100% genome completeness, and a sequencing coverage of 217.0×. Functional annotation, performed using the NCBI Prokaryotic Genome Annotation Pipeline (PGAP) version 6.11, identified 4429 coding sequences (CDSs). To evaluate the metabolic potential of the isolate, biosynthetic gene clusters (BGCs) associated with secondary metabolites were characterized using the antibiotics and secondary metabolite analysis shell (antiSMASH), version 8.0.4. This analysis revealed a diverse secondary metabolome consisting of 23 BGCs. These clusters were further assessed for their potential to produce antimicrobial compounds, underscoring the strain’s functional capacity for specialized metabolite biosynthesis (Figure 6a). The identified BGCs of the FNL62 genome include several classes of non-ribosomal peptide synthetases (NRPS) and polyketide synthases (PKS), appearing both as standalone clusters and hybrid architectures such as transAT-PKS/NRPS, NRPS/T1PKS, and NRPS-like/NRPS complexes. Additionally, the genome harbors clusters for ribosomally synthesized and post-translationally modified peptides (RiPPs), including azole-containing RiPPs and RiPP-like sequences. The genome also contains Type III PKS (T3PKS), terpene precursors, phosphonates, cyclic lactone autoinducers and rathipeptides. Notably, specialized metal-acquisition clusters, including NRPS-independent (NI) siderophores and NRP metallophores, were also identified, along with RRE-containing regions that may play a role in the regulation or modification of these specialized metabolites. Among the 23 annotated BGCs, 12, 6, and 5 were identified as novel, low-confidence, and high-confidence predictions, respectively.

Taxonomic identification of the FNL62 strain was performed by assessing genomic relatedness using the Type (Strain) Genome Server (TYGS). Pairwise genomic comparisons were executed using the Genome BLAST Distance Phylogeny (GBDP) method to verify the relationship of the FNL62 strain against the TYGS database. The GBDP analysis yielded similarity scores of 76.9% (95% CI: 72.9–80.4%), 38.0% (95% CI: 35.5–40.5%), and 67.9% (95% CI: 64.5–71.1%) for distance formulas d_0_, d_4_, and d_6_, respectively. These values, falling significantly below the thresholds for species classification, indicate that while the FNL62 strain is phylogenetically closest to *Brevibacillus laterosporus* DSM 25, it likely represents a novel species within the genus *Brevibacillus* (Figure 6b). The FNL62 isolate was designated *Brevibacillus* sp. FNL_62, and its genomic sequence has been deposited in the NCBI database under the accession number JBYRUE000000000.

To further support this taxonomic placement, the average nucleotide identity (ANI) across orthologous genes was calculated using FastANI, an alignment-free computational tool. Originally developed for bacterial species identification, this method was utilized to establish rigorous taxonomic boundaries for the isolates. In accordance with established genomic standards, assemblies exhibiting an ANI value ≥ 95% were considered to belong to the same species. Conversely, those yielding pairwise values between 80% and 95% were classified as different species, while values dropping below 80% designated the isolates as distinct genera [14]. Analysis of the *Brevibacillus* sp. FNL62 strain revealed significant genomic gaps, providing robust evidence for its taxonomic separation. Specifically, the highest ANI value was observed between the orthologous genomes of *Brevibacillus* sp. FNL62 and *Brevibacillus laterosporus* DSM 25, which shared 89.39% similarity across the entire orthologous mapping. This correspondence involved 1499 orthologous matches out of a possible 1800 sequences from the *B. laterosporus* DSM 25 genome.

A functional gene comparison between *Brevibacillus* sp. FNL62 and *B. laterosporus* DSM 25 was conducted using PGAP-annotated CDS data to identify conserved functional genes and cellular machinery. Homologous gene matching was evaluated using the Jaccard similarity index, and the best-matching reciprocal CDS pairs were visualized on a circular synteny map. A total of 4429 CDSs from *Brevibacillus* sp. FNL62 (outer circle) were mapped against the *B. laterosporus* DSM 25 genome (inner circle). The results revealed highly distinct functional gene profiles, supporting the classification of *Brevibacillus* sp. FNL62 as a novel species (Figure 6c). To further investigate this divergence, the matched genes were categorized by their degree of similarity. Only 177 CDSs exhibited high to moderate similarity (>50% similarity). This highly conserved subset predominantly consisted of essential housekeeping genes, specifically those involved in ribosomal function and protein synthesis. In contrast, the vast majority of the CDSs (4252 out of 4429) fell into the low or very low similarity tiers (<50% similarity). This overwhelming proportion of divergent genes highlights a significant functional distance from *B. laterosporus* DSM 25, strongly reinforcing the novel species designation. Meanwhile, the high and moderate similarity observed among essential housekeeping genes confirms that strain FNL62 correctly belongs to the *Brevibacillus* genus. Conversely, the massive subset of genes exhibiting low and very low similarity (<50%) represents a vast reservoir of uncharacterized genetic material, highlighting a critical opportunity for future functional gene exploration and discovery (Figure 6d).

### 2.8. Structural Evolution and Biophysical Trajectory Metrics of FNL62-AMP Membrane Disruption

**Figure 7:**
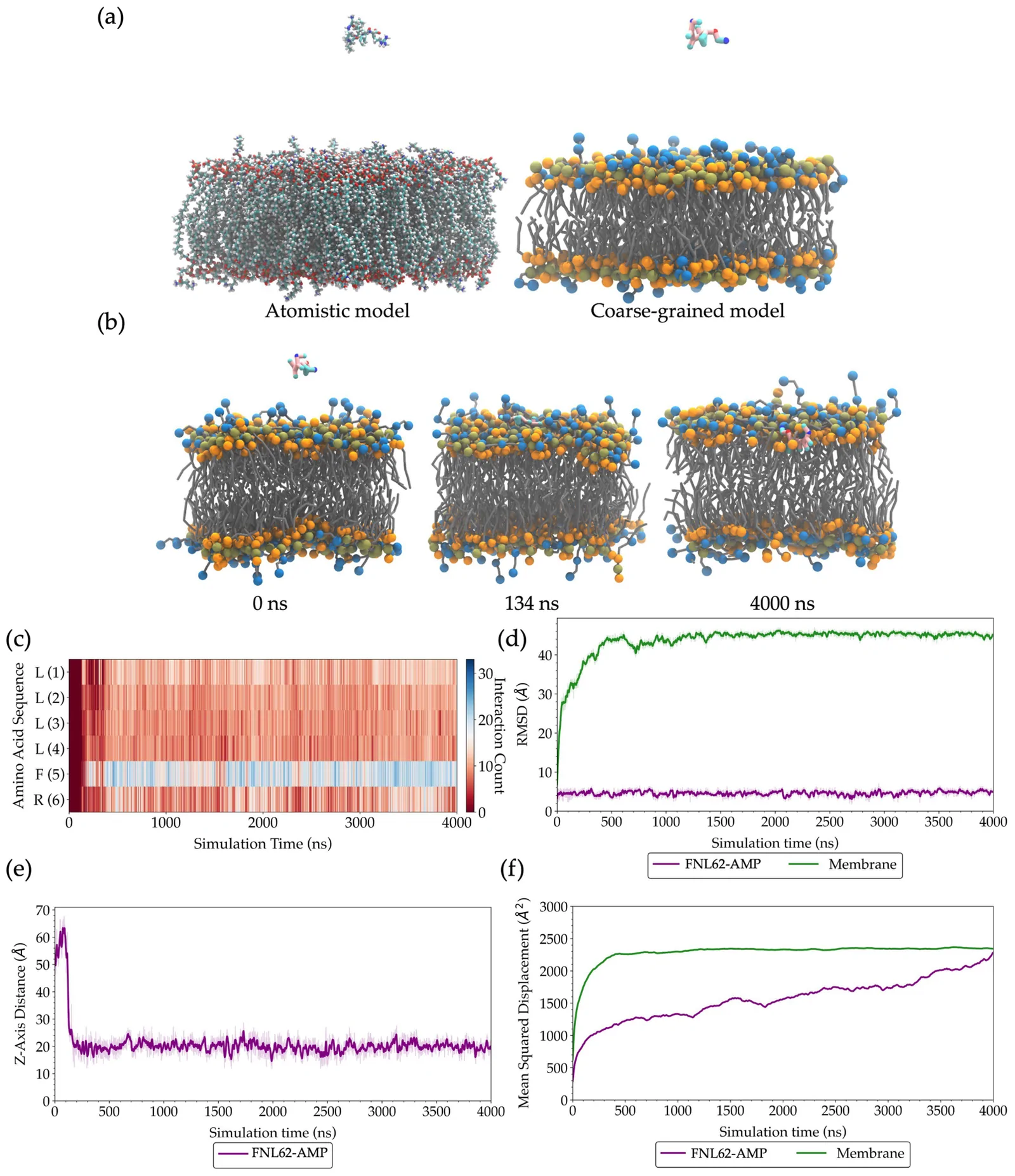
Molecular dynamics (MD) simulation trajectory and biophysical metrics tracking the FNL62-AMP/membrane system over a 4000 ns timeline. The atomistic model, represented using a CPK coloring style, was converted into coarse-grained beads parameterized according to the MARTINI force field (**a**). Representative simulation snapshots showing the peptide configuration in the aqueous phase at the start of the simulation (0 ns), at initial membrane adsorption (134 ns), and upon final interfacial insertion into the upper lipid leaflet (4000 ns) (**b**). Evolutionary contact heatmap tracking the interaction frequency between individual amino acid residues (*y*-axis) and the membrane surface over time (*x*-axis) (**c**). Root-mean-square deviation (RMSD) profiles of the lipid membrane and FNL62-AMP as a function of simulation time (**d**). Time-dependent center-of-mass (COM) *z*-axis distance of FNL62-AMP relative to the membrane center (**e**). Mean squared displacement (MSD) plots tracking the translational mobility of both the lipid matrix and FNL62-AMP over the trajectory (**f**). In the peptide color scheme, cyan represents the hydrophobic leucine and phenylalanine residues, blue denotes the positively charged arginine residues and N-terminal amine moieties, red indicates the negatively charged C-terminal carboxylic moieties, and pink highlights the polar peptide backbone. For the phospholipid model, the olive-green headgroups correspond to the negatively charged phosphate moieties, the orange beads represent the polar hydroxyl and glycerol regions, and the blue beads indicate the positively charged lysyl moieties. Finally, the gray sections denote the hydrophobic lipid tails. The data in (**d**–**f**) are presented as a moving average (window size = 50), with the shaded regions representing the raw values.

Initial atomistic models of both the peptide and the membrane were parameterized using the CHARMM36 all-atom force field prior to their conversion into coarse-grained models governed by the MARTINI force field (Figure 7a). Trajectory data from the MD simulations revealed a distinct conformational evolution of FNL62-AMP over the 4000 ns simulation period. The peptide initially associated with the membrane surface at 134 ns, subsequently undergoing a spontaneous structural transition from an interfacial adsorbed state to an interfacial insertion within the lipid headgroup region (Figure 7b). The contact heatmap depicts the spatiotemporal interaction frequency between individual amino acid residues and the bacterial membrane to reveal the primary anchoring sequence. Across the 4000 ns simulation trajectory, initial membrane contact was established by the arginine (R6) and N-terminal leucine (L1) residues via electrostatic interactions. The anchoring of these two primary residues subsequently facilitated the binding of the remaining peptide to the membrane surface. During overall peptide penetration, the phenylalanine residue (F5) recorded the highest interaction count, clearly demonstrating the critical role of its hydrophobic aromatic ring during the membrane-adsorption and membrane-binding phases (Figure 7c). The root-mean-square deviation (RMSD) of the membrane and FNL62-AMP was monitored across the entire 4000 ns trajectory. The RMSD of the membrane rapidly increased to about 45 Å at 500 ns, after which the RMSD remained constant until 4000 ns, while the FNL62-AMP RMSD remained stable at about 5 Å across the entire simulation (Figure 7d).

Analysis of the center-of-mass (COM) *z*-axis distance further clarifies the kinetics of membrane adsorption and insertion (Figure 7e). Between 0 and 100 ns, the high z-distance of approximately 60 Å confirms that the peptide remained entirely within the aqueous phase, diffusing freely in bulk water. Following this initial phase, a progressive decrease in the *z*-axis distance signifies the onset of a continuous attractive interaction between the bacterial membrane and FNL62-AMP, matching the initial contact visually captured in the representative snapshot at 134 ns. Subsequently, a rapid decline in the z-distance occurred for approximately 200 ns before stabilizing at a steady plateau of roughly 20 Å until 4000 ns. This stabilization at a 20-Å coordinate corresponds to the lipid headgroup region, which is consistent with the snapshot at 4000 ns.

To evaluate translational mobility and system stability, the mean squared displacement (MSD) of both the lipid matrix and FNL62-AMP was tracked across the trajectory (Figure 7f). The MSD values for the phospholipid bilayer remained remarkably flat and stable throughout the trajectory, confirming the tightly packed structural integrity and baseline rigidity of the bacterial membrane prior to peptide insertion. In contrast, the translational dynamics of the peptide captured distinct environmental transitions. Throughout the entire 0- to 4000 ns timeframe, which includes its initial diffusion in the bulk aqueous phase, the peptide’s MSD exhibited a gradual, linear increase over time. This regime reflects a period of unrestricted peptide movement on the membrane surface [15].

### 2.9. Self-Assembly Kinetics and Structural Evolution of the FNL62-AMP Macro-Aggregate

**Figure 8:**
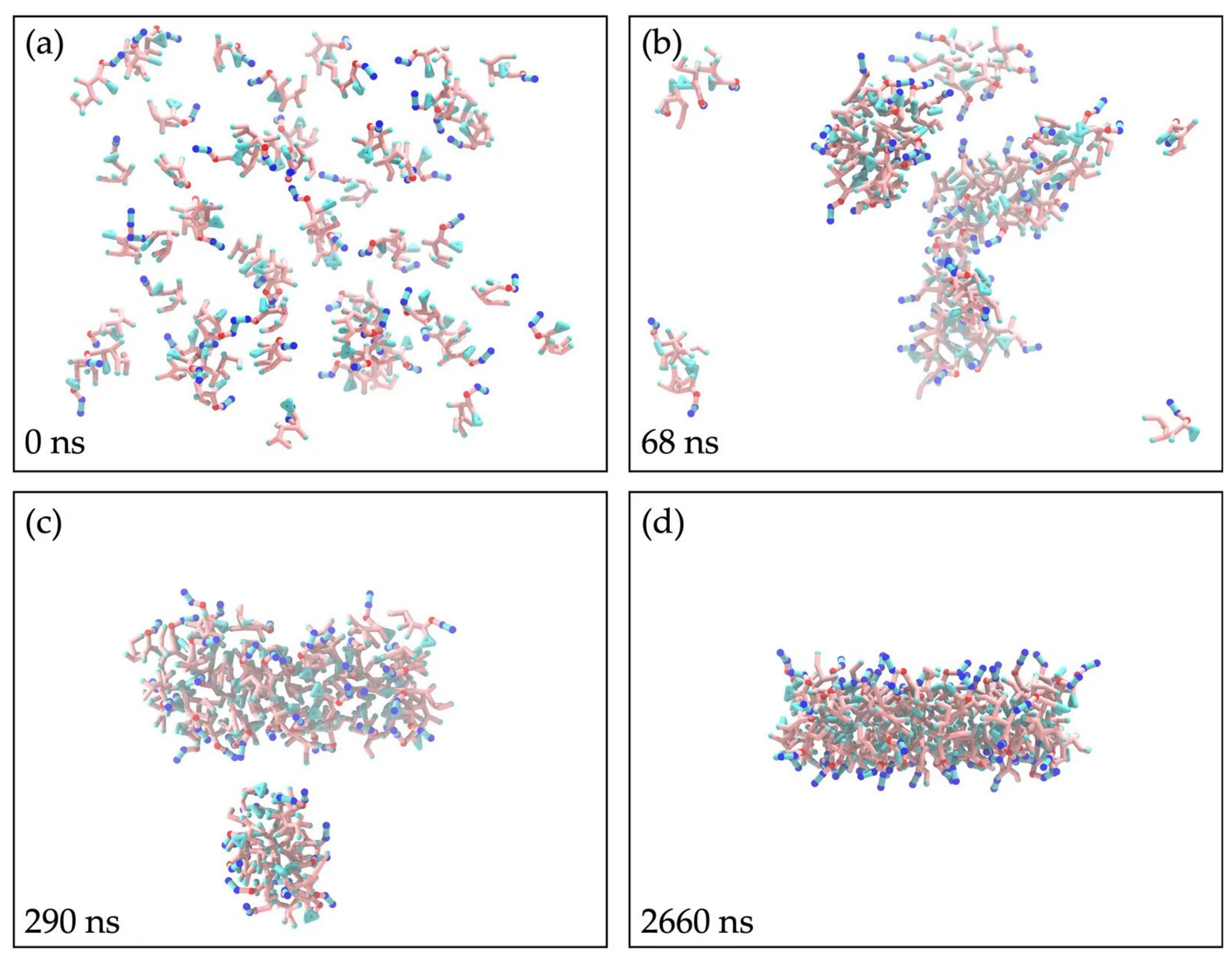
Self-aggregation timeline of 64 FNL62-AMP molecules in bulk water. Initial non-homogeneous mixture of isolated monomers and transient oligomers at 0 ns (**a**). Hydrophobic collapse into packed configurations by 68 ns (**b**). Segregation of the system into two distinct sub-aggregates at 290 ns (**c**). Final coalescence into a unified 64-peptide aggregate by 2660 ns (**d**). The color scheme applied to the peptide is defined in Figure 7.

The primary sequence of FNL62-AMP was determined to be LLLLFR. This hexapeptide is dominantly hydrophobic, driven by the nonpolar, aliphatic leucine (L) residues and the nonpolar, aromatic phenylalanine (F) residue, while the terminal arginine (R) introduces a net positive charge (+1) at physiological pH. In the aqueous coarse-grained MD simulations, the 64 FNL62-AMP molecules rapidly self-assembled into a well-defined core–shell micellar architecture. This structural organization was driven by the peptide’s distinct amphipathic character; its nonpolar segments collapsed inward to form a packed hydrophobic core, while the positively charged arginine residues oriented outward toward the solvent to maximize favorable electrostatic interactions with the aqueous phase (Figure 8). The time-resolved self-aggregation behavior of the 64 FNL62-AMP molecules within the aqueous phase was captured across the simulation trajectory. At the onset of the trajectory (0 ns), the peptide population is structurally non-homogeneous, consisting of a mixture of scattered transient oligomers and completely isolated monomers. During initial equilibration within the bulk aqueous phase, these scattered monomers rapidly underwent hydrophobic collapse to minimize solvent exposure. This prompt self-assembly yielded highly organized, micelle-like structures that effectively buried the nonpolar hydrophobic aromatic side chains within a shielded core while orienting the highly cationic residues outward toward the water molecules. This aggregation process progressed cooperatively over time, producing a denser and more tightly packed molecular assembly by 68 ns. By 290 ns, the system dynamically segregated into two distinct, well-defined macromolecular sub-aggregates. These separate entities underwent a final, comprehensive consolidation by 2660 ns, merging into a singular, unified 64-peptide aggregate.

**Figure 9:**
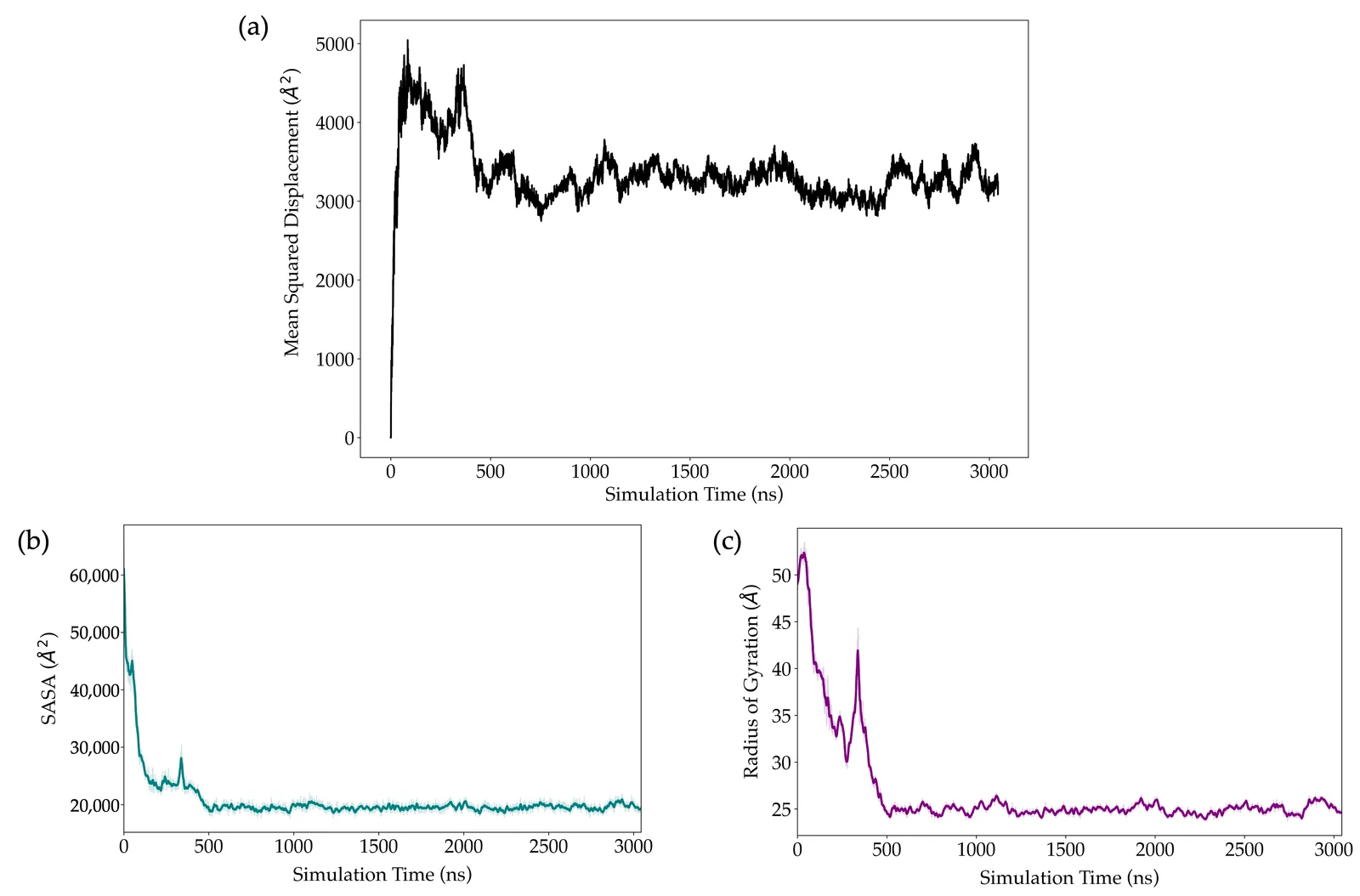
Structural and kinetic profiles of the 64-peptide aggregate system. Mean squared displacement (MSD) profile of the 64-peptide aggregate (**a**). Evolution of the solvent-accessible surface area (SASA) during self-assembly (**b**). Structural compactness of the aggregate based on the radius of gyration (*R_g_*) trajectory (**c**). The data in (**b**,**c**) are presented as a moving average (window size = 50), with the shaded regions representing the raw values.

To evaluate these assembly kinetics, the MSD profile of the aggregate formed by 64 FNL62-AMP molecules was monitored across the entire simulation timeline. During the initial phase of the trajectory, the MSD values increased sharply and linearly, indicating that the unconstrained AMP molecules diffused and displaced rapidly through the bulk aqueous phase. Following this highly mobile phase, the MSD curve transitioned into a stable, constant plateau. This flattening of the MSD slope denoted a significant restriction in translational mobility, signifying that the individual AMP molecules had locked in place relative to one another. Biophysically, this behavior confirmed the completion of hydrophobic collapse and the successful formation of a stable, tightly packed multi-peptide aggregate (Figure 9a). The total solvent-accessible surface area (SASA) dropped sharply at the beginning of the simulation, matching the exact time when the peptides first began to associate. This surface area continued to decline as smaller clusters steadily combined into larger structures, shielding the internal residues of the peptides from the surrounding water. The value eventually flattened out at a stable baseline of approximately 20,000 Å^2^, confirming that the final 64-peptide aggregate had reached a steady and balanced structure (Figure 9b). The radius of gyration (*R_g_*) analysis was conducted to evaluate the structural compactness of the system [16]. The *R_g_* profile demonstrated that FNL62-AMP underwent a significant structural transition during the trajectory. The *R_g_* value decreased from an initial 50 Å and ultimately stabilized at 25 Å, indicating the transition into a highly compact conformation. This substantial reduction in global molecular dimensions directly confirmed the successful formation of a stable, tightly bound 64-peptide aggregate (Figure 9c). To support the results of the simulation, in vitro biophysical assays were conducted to physically monitor the self-assembly of FNL62-AMP in solution. Intrinsic fluorescence spectroscopy was used to track the self-assembly behavior across a concentration gradient (1×, 2×, 4×, and 8× MIC). The results revealed a strict concentration-dependent increase in intrinsic fluorescence intensity, scaling proportionally from 7996.33 ± 220.58 at 1× MIC to 14,526.00 ± 190.37 at 2× MIC, 28,914.67 ± 424.65 at 4× MIC, and reaching 56,923.67 ± 1631.23 at 8× MIC. Furthermore, dynamic light scattering (DLS) was employed to quantify the physical size of these assemblies in the aqueous phase. The average hydrodynamic diameters measured 430.20 ± 100.81 nm at 1× MIC, 354.60 ± 104.11 nm at 2× MIC, 463.80 ± 39.85 nm at 4× MIC, and 439.20 ± 66.21 nm at 8× MIC. Crucially, although the intrinsic fluorescence intensity increased in a concentration-dependent manner, indicating an increasing total mass of aggregated peptide in the solution, the hydrodynamic size of the structures remained relatively steady. This steady DLS profile indicates that rather than growing indefinitely into unstructured, infinite clumps, the peptides spontaneously organize into structurally stable, pre-defined macro-aggregates that maintain a specific size threshold. Ultimately, these in vitro results provide robust preliminary support bridging the molecular dynamics simulations with physical experiments; however, the comprehensive characterization of this peptide aggregation and its precise biophysical functions will be further investigated in future studies.

### 2.10. Molecular Dynamics Simulations of Multi-Peptide Aggregation and Membrane Insertion

**Figure 10:**
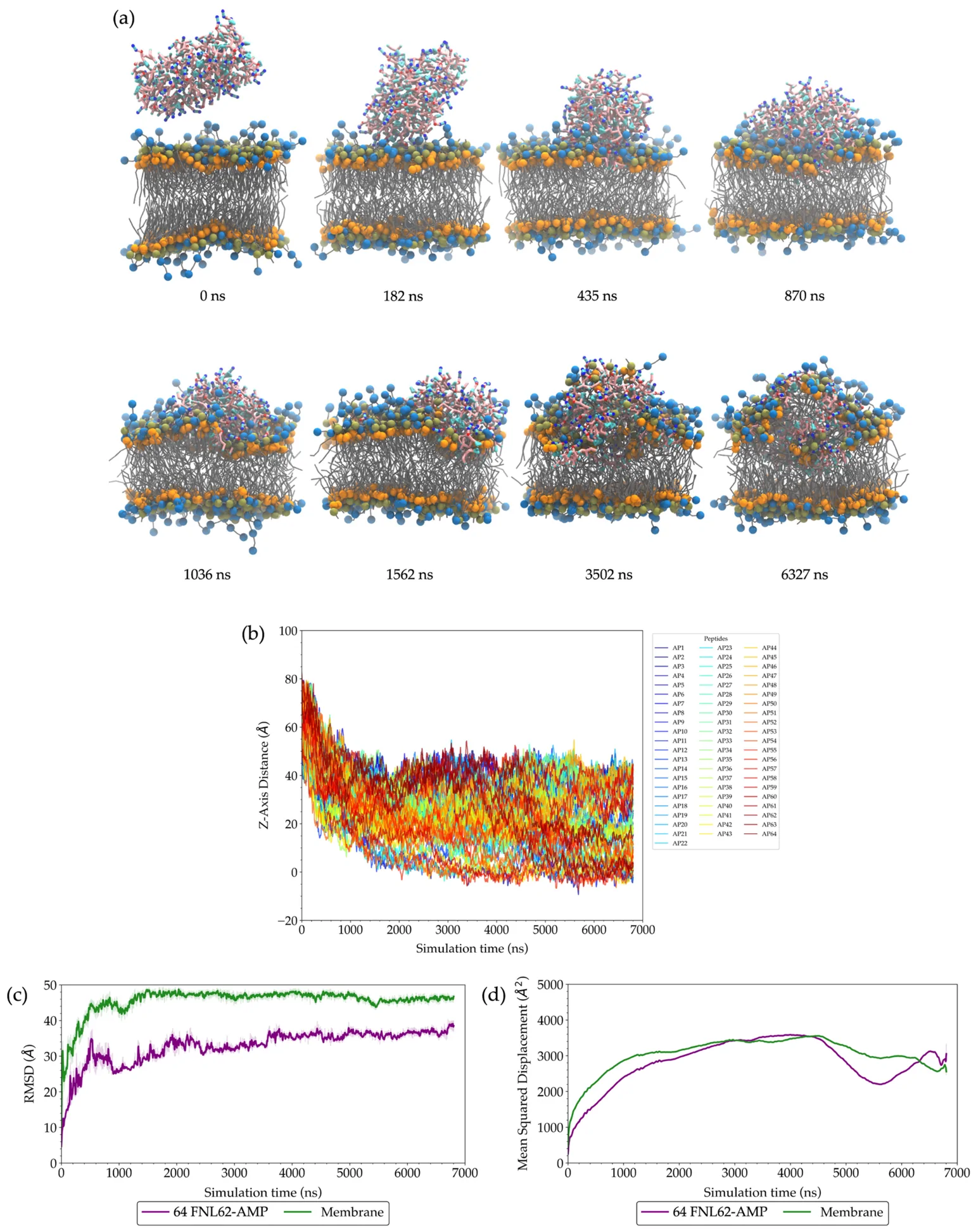
Molecular dynamics (MD) simulation trajectory of a 64-peptide aggregate demonstrating multi-peptide membrane-lytic activity over 6327 ns (**a**). Penetration of the 64-peptide aggregate (AP1–AP64) into the membrane, illustrated by the center-of-mass (COM) distance between the peptide aggregate and the membrane (**b**). Root-mean-square deviation (RMSD) (**c**) and mean squared displacement (MSD) profiles (**d**) of the 64-peptide aggregate and the membrane throughout the simulation. The color scheme applied to the peptide and membrane models is defined in Figure 7. The data in (**b**–**d**) are presented as a moving average (window size = 50), with the shaded regions representing the raw values.

CG-MD simulations were further performed using a mimetic *S. aureus* bilayer composed of 1,2-dipalmitoyl-sn-glycero-3-phosphoglycerol (DPPG; 40%), lysyl-DPPG (52%), and tetramyristoyl cardiolipin (8%) [17]. This specific lipid composition reflects the complex anionic environment and net charge density characteristic of the staphylococcal cell envelope. Within this membrane model, the positively charged arginine residues mediated the initial electrostatic interactions with the phospholipid headgroup region of the bacterial membrane. Concurrently, the aggregated hydrophobic core of the AMP was inserted directly into the phospholipid bilayer, disrupting the membrane matrix. To elucidate how this macro-assembly drives membrane disruption, the fully consolidated 64-peptide aggregate was simulated alongside a representative bacterial membrane over an extended 6327 ns trajectory (Figure 10a). This organized peptide aggregate initially localized at the membrane interface at 182 ns, establishing direct electrostatic contacts with both the anionic lipid phosphate centers (green headgroups) and the positively charged domains of the lysyl-DPPG (blue) matrices. Following this interfacial accumulation, the cohesive micellar cluster progressively partitioned deeper into the core, initiating continuous insertion into the hydrophobic lipid bilayer starting at 435 ns. The depth of the peptide insertion increased steadily throughout the remainder of the simulation, culminating in bilayer disruption and membrane rupture by the 6327 ns mark. These computational coordinates provide direct confirmation of a membrane-lytic mechanism propagated by the peptide aggregate. The enhanced penetration of the aggregated peptide was evidenced by the *z*-axis distance between the aggregate and the membrane center dropping below 0 Å, indicating that portions of the peptide aggregate reached the hydrophobic core of the membrane (Figure 10b).

During the first 1000 ns of the simulation, fluctuations and rapid increases in the RMSD for both the peptide and the membrane indicated progressive interactions. A slight reduction in RMSD around 1000 ns suggested that the initial binding phase was complete; the resulting complex restricted the molecular mobility of both components, leading to the observed RMSD decrease. After 1000 ns, the membrane RMSD slightly increased before reaching a plateau, signifying that the bound peptide aggregate restricted the movement of lipid components and reduced membrane fluidity. In contrast, the peptide RMSD continued to increase, indicating that the monomers were undergoing structural rearrangement to minimize their energy upon binding. The peptide RMSD subsequently plateaued at approximately 3500 ns, demonstrating that the aggregate was tightly bound to the upper leaflet, which severely restricted further conformational changes (Figure 10c). MSD analysis corroborated the RMSD findings regarding the disruption of membrane fluidity. While intact lipid bilayers typically constrain lateral lipid motility within a restricted area to reach a stable plateau, peptide interactions severely interfere with this movement. This interference occasionally induces pore formation and membrane curvature, ultimately leading to bilayer disruption. By 4000 ns, both the peptide and the membrane reached a plateau phase accompanied by a slight reduction in MSD, indicating that the binding interaction had reached equilibrium and molecular diffusion was highly restricted (Figure 10d).

### 2.11. Membrane Topography Analysis and Localized Bilayer Deformation Induced by FNL62-AMP Aggregate

**Figure 11:**
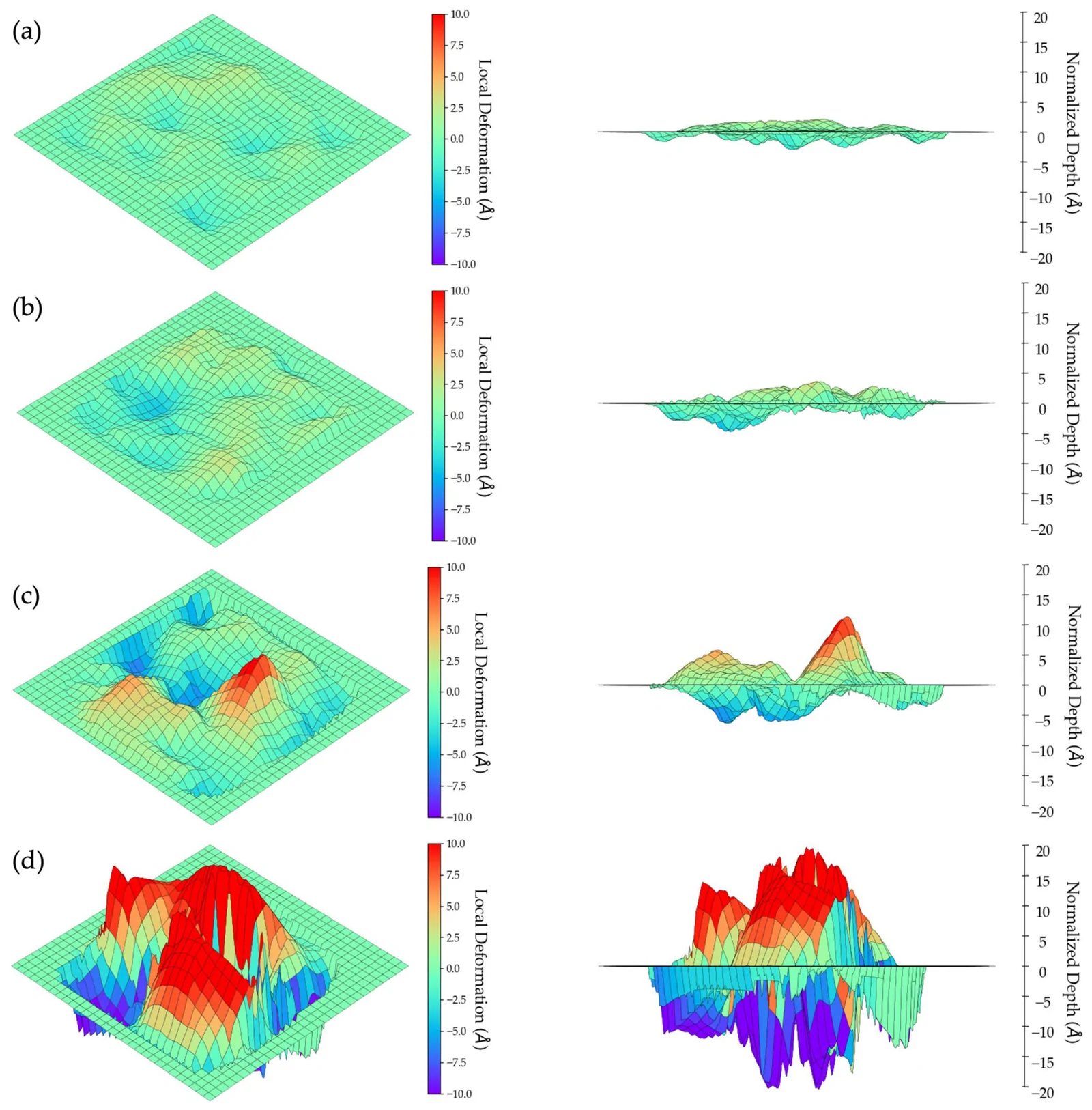
Quantitative analysis of localized membrane deformation and normalized bilayer depth profiles. Topographic local deformation maps of the bacterial membrane interacting with a 64-peptide aggregate at key simulation intervals: 0 ns (**a**), 182 ns (**b**), 1036 ns (**c**), and 6327 ns (**d**).

To measure the physical disruption caused by the 64-peptide aggregate, variations in the local membrane topography and normalized insertion depth were monitored at distinct simulation intervals (182 ns, 1036 ns, and 6327 ns). The initial state of the system at 0 ns represented the pre-binding membrane, serving as the control baseline (Figure 11a). During the initial binding phase at 182 ns, surface adsorption and early penetration into the upper phospholipid layer triggered noticeable localized matrix distortion. Regions displaying a negative deviation (values below 0.0 Å) revealed a downward dimpling of the membrane toward its central core, tracking the inward push of the advancing peptide mass. Concurrently, neighboring zones exhibited a positive deviation (values above 0.0 Å), capturing the upward crowning of lipid headgroups being pushed aside by the bulky peptide aggregate (Figure 11b).

By the 1036 ns mark, the 64-peptide aggregate had successfully advanced into the inner hydrophobic zone of the bacterial bilayer (Figure 11c). This topographic warping became severely amplified by the 6327 ns endpoint, at which point the 64-peptide aggregate had achieved complete localization within the center of the lipid bilayer. The marked deepening of these local surface depressions demonstrated that this large, cooperative 64-peptide aggregate forced a severe physical rearrangement of the surrounding lipids, resulting in the irreversible collapse of the membrane structure (Figure 11d).

## 3. Discussion

The production of antibacterial compounds by the FNL62 isolate was initiated during the late exponential phase (16–20 h), a kinetic profile commonly triggered by nutrient depletion or metabolic shifts within the culture medium [18]. The isolate demonstrated sustained antibacterial spectrum efficacy against Gram-positive pathogens (*S. aureus* and MRSA) from 16 h through the end of the 168 h incubation period. In contrast, its inhibitory action against the Gram-negative *K. pneumoniae* was highly time-specific, appearing strictly within a narrow 24–72 h window. This divergence indicates that the isolate may synthesize structurally distinct antibacterial compounds at different metabolic stages, or alternatively, that the specific molecular configurations responsible for anti-*K. pneumoniae* activity are chemically unstable and undergo rapid degradation or enzymatic inactivation after 72 h in the fermentation broth. Therefore, elucidating the time-dependent metabolic profile of the FNL62 isolate warrants further investigation to identify distinct antibacterial compounds active against a broader spectrum of Gram-positive and Gram-negative pathogens. Furthermore, it must be noted that the current study evaluated the efficacy of the purified FNL62-AMP strictly against *S. aureus* and MRSA isolates. The absolute activity of the purified peptide has not yet been established against genetically diverse clinical isolates or Gram-negative species. Future investigations will prioritize standard MIC/MBC determinations of the purified FNL62-AMP against a significantly expanded pathogen panel to fully define its antimicrobial spectrum. The purification of FNL62-AMP was achieved through an orthogonal purification strategy leveraging distinct physicochemical properties, sequentially employing salting out, ion-exchange chromatography, and reverse-phase chromatography, including solvent exchange with dialysis. As highlighted in the recent literature, successfully extracting potent and highly pure AMPs from complex bacterial cultures consistently requires multiple, specialized purification stages [19]. The short AMP sequence with the ability to form micelles or aggregate by self-assembly could contribute to the retention ability and be achieved by dialysis with a larger MWCO than its molecular weight [20]. Following purification, de novo sequencing is the gold standard for elucidating the primary structure of novel short peptides. However, bacterial non-ribosomal peptide synthetase (NRPS) pathways frequently introduce complex structural modifications, including the incorporation of unnatural amino acids and altered stereochemistry [21,22]. Because standard LC-MS/MS cannot readily differentiate between isobaric residues such as leucine and isoleucine, resolve chiral stereochemistry, or confirm exact N- and C-terminal states, the absolute structural elucidation of FNL62-AMP remains a limitation of the current study. Fully defining these structural nuances will be the focus of future investigations employing orthogonal analytical techniques alongside ongoing lead optimization efforts [23,24].

The killing kinetics of the purified FNL62-AMP followed a clear, concentration-dependent mechanism. At lower doses (1× and 2× MIC), the peptide managed to arrest growth but failed to achieve complete microbial clearance within the initial timeframe. Conversely, higher doses (4× and 8× MIC) induced rapid bactericidal action, culminating in complete microbial eradication by 12 h and 8 h, respectively. This acute, destructive capability is fundamentally driven by direct physical disruption of the bacterial envelope. The membrane-lytic pathway of FNL62-AMP is visually confirmed by SEM imaging showing cellular deformation, which directly correlates with the in vitro SYTOX Green permeabilization assays. Including, the result of time-kill kinetics demonstrated that absolute bactericidal action requires prolonged exposure to reach a critical damage threshold; the 16 h timepoint was specifically selected to capture the terminal morphological collapse of the cells. Thus, these SEM images represent late-stage lysis resulting from cumulative peptide-induced membrane damage, while the primary, immediate membrane destabilization events are reflected in the rapid SYTOX Green uptake and molecular dynamics simulations. Exposure to the peptide triggers a sharp, immediate surge in SYTOX Green fluorescence, confirming the instantaneous loss of bilayer barrier function. When these biological findings are integrated with computational COM trajectory analyses—which show the peptide rapidly partitioning and stabilizing precisely at the 20-Å lipid headgroup–tail boundary—the evidence firmly establishes that FNL62-AMP kills via a direct, physical membrane-lytic mechanism. This observed antimicrobial activity is consistent with previous reports on *Brevibacillus laterosporus* TSA31-5, which produces several AMPs active against MRSA (MIC range of 1–8 μg/mL) that also induce bacterial membrane disruption [25].

Similar to established AMPs in the bacitracin family, the short peptide produced by this *Bacillus* species demonstrated high tolerance across several stress conditions [26]. The physical nature of this mechanism proposes the suitable resilience observed in FNL62-AMP. The peptide displays remarkable thermal stability, preserving full anti-MRSA potency even after 6 h of exposure to a high thermal stress of 80 °C. FNL62-AMP exhibits optimal activity within a near-neutral pH range (pH 6–8). Exposure to extreme acidic or alkaline conditions likely alters the peptide’s ionization state and net charge, which are crucial factors for mediating its interaction with the bacterial membrane. Furthermore, its enzymatic degradation profile reveals encouraging selectivity. The experimental proteolytic profile of FNL62-AMP, exhibiting maximum degradation by α-chymotrypsin, moderate degradation by proteinase K, and high resistance to trypsin, is highly consistent with the LLLLFR sequence. This specific digestion profile can be attributed to several biochemical and structural factors. α-Chymotrypsin acts as a highly specialized endopeptidase with a strong catalytic preference for aromatic (phenylalanine) and large hydrophobic (leucine) residues, leading to rapid, multiple-site cleavage of the peptide backbone. While proteinase K possesses a broad-spectrum specificity that encompasses these same hydrophobic targets, the highly specialized kinetics of α-chymotrypsin likely drive the more aggressive degradation observed in vitro. Furthermore, the propensity of FNL62-AMP to self-assemble into hydrophobic macro-aggregates induces selective steric hindrance. In this aggregated state, α-chymotrypsin favorably targets the more exposed phenylalanine and arginine residues located on the exterior of the macro-aggregates. In contrast, proteinase K favors the aliphatic and aromatic residues embedded deep within the tightly packed hydrophobic core, rendering them significantly harder to access and cleave. Conversely, the singular basic arginine residue located at the extreme C-terminus deprives trypsin of the internal cleavage sites required for fragmentation, endowing the peptide with remarkable tryptic stability [27]. Furthermore, the susceptibility of FNL62-AMP to these specific proteolytic enzymes strongly suggests that its sequence consists of standard L-amino acids. If the peptide contained D-amino acid stereoisomers, it would typically exhibit pronounced resistance to such enzymatic degradation [28,29]. Although the present study provides robust biochemical evidence supporting the proposed LLLLFR sequence, including its likely L-amino acid composition indicated by specific proteolytic degradation profiles, a notable limitation is the exclusive reliance on the naturally isolated FNL62-AMP. To achieve absolute structural validation, the chemical synthesis of the peptide must be conducted. Therefore, future studies will prioritize this chemical synthesis, alongside rigorous spectrometric and functional comparisons, to definitively investigate the L- or D-stereochemistry and fully authenticate the peptide’s exact sequence configuration.

Co-incubation studies with structurally diverse surfactants revealed critical, charge-dependent interaction profiles. The addition of SDS caused an acute decrease in antimicrobial efficacy. This is primarily driven by intense electrostatic attraction and charge neutralization between the negatively charged headgroups of the anionic surfactant and the positively charged cationic residues of the peptide [30]. This tight electrostatic complexation shields the peptide’s active charges, preventing them from binding to the anionic bacterial cell wall. Triton X-100, a nonionic surfactant, did not alter the native antibacterial activity of FNL62-AMP. This observation aligns with the existing literature indicating that co-formulating a peptide with a nonionic surfactant preserves its intrinsic efficacy, a phenomenon attributed to the inherently weak physical interactions between the uncharged surfactant headgroups and the peptide backbone [30]. While it is well known that most cationic surfactants exhibit intrinsic antimicrobial properties at specific concentrations, the combination of CTAB and FNL62-AMP unexpectedly decreased the overall antibacterial activity compared to either agent applied alone. This antagonistic effect could stem from steric hindrance or structural interference, where the cationic surfactant chains disrupt or misfold the active conformation of FNL62-AMP, preventing efficient membrane insertion and peptide aggregation. Interestingly, this finding contrasts with certain literature, such as the formulation of the G3 peptide with CTAB, which reported enhanced synergistic antimicrobial activity [30], highlighting that the compatibility of cationic surfactants is highly sensitive to the specific primary sequence of the target peptide. This discrepancy highlights that the compatibility of cationic surfactants is highly sensitive to the specific primary sequence of the target peptide. Consequently, this unpredictability underscores the need for future studies to systematically screen the peptide within complex, multicomponent formulation matrices to ensure its intrinsic bioactivity is fully preserved in practical applications.

While the agar well diffusion assay was used in stability experiments to provide valuable preliminary insights into the functional resilience of FNL62-AMP under various environmental stressors, the inherent limitations of this method must be acknowledged. The observed residual activity reflects the net functional bioactivity of the peptide within the agar matrix, which can be confounded by variables such as altered diffusion rates, pH, ionic strength, and the intrinsic antimicrobial or aggregative properties of the tested surfactants (e.g., SDS, CTAB, and Triton X-100) [31,32]. Therefore, these data serve as a preliminary functional screening to guide initial handling, storage, and early-stage formulation strategies, rather than an absolute measure of molecular stability. To definitively establish the structural integrity and long-term stability of FNL62-AMP, future comprehensive formulation studies will utilize reversed-phase high-performance liquid chromatography (RP-HPLC) and liquid chromatography-mass spectrometry (LC-MS) for direct molecular quantification, coupled with standard broth microdilution MIC re-determinations following the neutralization of stress agents.

Genomic analysis of the FNL62 isolate provides definitive evidence of a novel species classification within the genus *Brevibacillus*. In modern prokaryotic systematics, an ANI threshold of ≥95% and a digital DNA-DNA hybridization (dDDH) value of ≥70% serve as the standard boundaries for species demarcation. Because the FNL62 strain shares a maximum ANI of only 89.39% with its closest relative, *Brevibacillus laterosporus* DSM 25, it diverges unambiguously from established species lineages. This taxonomic separation is further validated by the TYGS results, where the calculated GBDP similarity scores fall dramatically short of the 70% dDDH gold standard. Together, these significant genomic gaps confirm that the FNL62 isolate represents a novel, distinct species.

The critical role that aromatic residues, specifically phenylalanine, play in anchoring AMPs to lipid bilayers provides a robust explanation for the computational findings. While the initial attraction between cationic AMPs and bacterial membranes is often driven by long-range electrostatic forces, hydrophobic aromatic residues are indispensable for providing a stable membrane anchor [33]. Biophysical scaling frameworks demonstrate that aromatic residues are uniquely favored at zwitterionic and anionic membrane interfaces, acting as the single most favorable contributors to deep bilayer insertion. The interfacial partitioning of any peptide into a membrane matrix is heavily dominated by the large, favorable energetic contributions of these bulky aromatic side chains. This thermodynamic preference provides a strong biophysical justification for the dominant role of the phenylalanine residue (F5) observed in the spatiotemporal contact heatmaps of FNL62-AMP [34]. A primary mechanism driving AMP self-assembly is the clustering of peptides into globular aggregates. These structures position their hydrophobic core internally while exposing a hydrophilic surface [35]. Upon interacting with the bilayer, these FNL62-AMP clusters undergo interfacial reorganization, orienting their hydrophobic facets toward the lipid matrix to facilitate the insertion process.

Individual amino acid residues exhibit distinct propensities toward this self-association. The aggregation is driven by the hydrophobic effect—specifically, a thermodynamic balance where attractive van der Waals interactions and solvent entropy drive clustering—while Coulombic repulsion pushes the monomers apart [36]. The equilibrium between these opposing forces dictates the maximum size of the aggregate, though exploring this specific saturation limit is beyond the scope of the present work. The literature indicates that aliphatic residues (Ile, Leu, Val, and Met) and aromatic residues (Phe, Tyr, and Trp) rapidly condense into dense clusters [36]. Because FNL62-AMP contains both Leu and Phe, it inherently provides the hydrophobic and aromatic interactions necessary to drive this self-association.

The trajectory-derived coordinates of the multi-peptide aggregate strongly correlate with established biophysical models of membrane lysis [37,38]. Specifically, the structural transitions executed by this assembly mirror the highly coordinated, multi-step cascade characteristic of a carpet- or detergent-like mechanism, as detailed in the literature for cationic peptides like BP100. This lytic process initiates with electrostatic interactions between the negatively charged membrane surface and the positively charged residues of the peptide aggregate. Following this initial surface targeting, the peptides undergo a distinct rotational reorientation termed a peptide flip, which prompts the immediate insertion of the peptide’s hydrophobic face directly into the nonpolar core of the lipid bilayer. This molecular rotation and subsequent insertion represent a crucial step within the broader carpet mechanism, as they accelerate localized lipid clustering and peptide aggregation across the outer leaflet. As these molecules accumulate, they drive the formation of interconnected, destabilized peptide–lipid patches. Ultimately, this progressive assembly disrupts the native lipid organization, leading to a rapid, detergent-like disintegration and the irreversible collapse of the membrane structure [39,40,41,42]. A similar mechanism of action against the simulated bacterial membrane is proposed for the 64-monomer FNL62-AMP aggregate. It is hypothesized that the localized micro-environment on the bacterial surface is accurately reflected by this computational model, where a critical mass threshold is reached through the progressive accumulation of hydrophobic, cationic peptides from the bulk solvent. The continuous decline of the total SASA highlights a steady consolidation process. Smaller intermediate clusters combine into larger structures, a behavior driven by the hydrophobic effect to shield nonpolar regions from the surrounding water. This observed kinetic profile aligns with established biophysical models of peptide aggregation, where initial assembly phases are characterized by a rapid reduction in solvent exposure as free AMP molecules establish their first intermolecular contacts. Furthermore, this SASA profile correlates perfectly with the *R_g_* trajectory, collectively confirming that the FNL62-AMP molecules have achieved a highly stable, self-aggregated macro-structure. Notably, rather than forming simple spherical micelles, our computational models indicate that these macro-aggregates adopt non-spherical, bilayer-like or cylindrical architectures. This specific geometric packing is driven by the peptide’s highly amphipathic structure; it allows the bulky hydrophobic residues to bury themselves efficiently within the core while ensuring the charged, cationic arginine tails remain oriented toward the aqueous interface. Furthermore, the delayed bactericidal kinetics observed in the time-kill and SEM assays are aligned with this localized accumulation model. Nevertheless, these MD findings must be considered strictly predictive. Furthermore, it is important to acknowledge that the molecular dynamics data presented herein, including the 4000 ns single-peptide trajectory, as well as the 2660 ns and 6327 ns aggregate simulations, were executed as single continuous production runs. While these preliminary simulations successfully propose a foundational model for peptide–membrane interactions, single-run trajectories inherently limit the statistical robustness of the conformational sampling. Therefore, future computational studies will incorporate systematic independent replicates with varied initial velocity seeds and spatial configurations to rigorously and statistically validate these interaction phenomena. To definitively validate this pre-aggregation and subsequent membrane disruption mechanism, real in vitro biophysical experiments are required. Rigorous evaluations must be incorporated with in silico simulation, the true aggregate size in actual solution must be quantified via dynamic light scattering (DLS), As demonstrated in GSLP models, DLS can effectively track concentration-dependent transitions, such as shifting from highly dispersed, heterogeneous assemblies at lower concentrations (e.g., 64 µM) to denser, regular predominant populations (ranging from 310 to 360 nm) at higher thresholds. Along with characterizing these physical aggregation states using DLS and complementary hydrophobic fluorescence probes (such as 1,8-ANS), the physical lipid-to-peptide ratios required for absolute bilayer rupture must be experimentally confirmed using liposome-based dye-leakage assays. Furthermore, microscopic imaging techniques such as transmission electron microscopy (TEM) were utilized to confirm whether these predominant populations further intertwine into extended nanonetworks, mimicking the self-assembled short nanoribbons observed in GSLPs. Validating the formation of these interconnected nanonetwork assemblies is critical, as this structural overlap not only enhances supramolecular integrity but also sterically shields the peptides from enzymatic degradation in physiological environments [43,44,45]. The in vitro biophysical assays provide empirical support for the self-assembly behavior predicted by the molecular dynamics simulations. The concentration-dependent increase in intrinsic fluorescence intensity, from 7996.33 ± 220.58 at 1× MIC to 56,923.67 ± 1631.23 at 8× MIC, indicates the progressive formation of hydrophobic microenvironments as the peptide concentration increases. This behavior is characteristic of self-assembling surfactant-like peptides; for example, the previous study reported similar concentration-dependent fluorescence enhancements associated with the progressive aggregation of WRWRWRWR ([WR]_4_) cyclic peptide nanostructures in solution [46]. Although the intrinsic fluorescence intensity increased linearly, indicating an increasing total mass of aggregated peptide in the solution, dynamic light scattering (DLS) profiles showed that the hydrodynamic size of these structures remained relatively stable across the tested concentrations (ranging between approximately 355 and 464 nm). This DLS profile suggests that rather than continuing to grow into larger, unstructured aggregates, the peptides assemble into structurally stable macro-aggregates within a specific size range. This observation is consistent with established models of supramolecular peptide assembly. The presence of defined size populations via DLS indicates that peptide building blocks form regular nanostructures rather than amorphous precipitates [47]. Furthermore, the physical dimensions and concentration-dependent behavior of the FNL62-AMP aggregates are comparable to the self-assembly characteristics reported for gemini surfactant-like peptides (GSLPs). The reported study showed peptide aggregate formation; at elevated concentrations, GSLPs transition into densely organized nanostructures with predominant sizes in the 310 to 361 nm range, driven by intermolecular noncovalent forces. The assembly of FNL62-AMP into macro-aggregates of a similar physical scale suggests that designing self-assembled nanosystems through specific amino acid arrangements offers a strategy to address the stability limitations of AMPs, particularly by sterically enhancing their resistance to protease degradation [45]. However, these in vitro results provide preliminary experimental data that correlate with the molecular dynamics simulations. By demonstrating that FNL62-AMP forms stable, size-restricted macro-aggregates, this study outlines a structural framework for the peptide. However, further characterization of this peptide aggregation and its biophysical functions is required in future studies.

The sequence of FNL62-AMP comprises 83% hydrophobic residues, which raises valid concerns regarding potential hemolysis, nonspecific mammalian cytotoxicity, and high serum protein binding [48]. Because peptide interactions with mammalian cell membranes are driven by physicochemical properties, predictive insights can be drawn from structural homology. Querying the APD6 database revealed that FNL62-AMP (LLLLFR) shares 50% sequence similarity with the de novo-designed peptide L4K2W^4^ (LLKWLLK). Notably, L4K2W^4^ exhibits antimicrobial activity against *S. aureus* (MIC of 25 µg/mL) that is comparable to that of FNL62-AMP. Furthermore, both peptides share analogous physicochemical parameters, including comparable net charges (+1 and +2, respectively), Wimley–White whole-residue hydrophobicities (−2.56 and −2.11 kcal/mol), hydrophobic residue contents (83% and 71%), and protein-binding potentials (Boman index: −1.29 and −1.55 kcal/mol). Importantly, L4K2W4 has been reported to exhibit less than 5% hemolytic activity at 200 μg/mL (relative to a 0.2% Triton X-100 control) [12]. The selective toxicity of AMPs toward bacterial targets over mammalian cells is well documented. This selectivity is primarily governed by electrostatic differences. The initial binding of an AMP to a membrane is driven by electrostatic attraction, after which the peptide’s hydrophobicity facilitates its penetration into the hydrophobic core of the lipid bilayer. Specifically, the net positive charge of AMP favors interactions with the negatively charged phospholipid bilayers characteristic of bacterial membranes (e.g., those containing phosphatidylglycerol, PG) rather than the neutral, zwitterionic phospholipids typical of mammalian cell membranes (e.g., those containing phosphatidylcholine, PC) [49,50,51]. Although this biophysical homology of selectivity suggests a favorable safety profile for FNL62-AMP, these remain strictly predictive parameters. Direct experimental determinations of the hemolytic dose (HC_50_), cytotoxic concentration (CC_50_), plasma stability, and solubility at biologically relevant concentrations remain mandatory prerequisites. Beyond these in vitro assessments, comprehensive in vivo toxicity evaluations, such as determining the therapeutic index and median lethal dose (LD_50_), are strictly required before FNL62-AMP can be advanced as a viable therapeutic lead.

## 4. Materials and Methods

### 4.1. Isolation of the Mangrove Bacterium

Mangrove sediment samples were collected from the forest floor in Nakhon Si Thammarat, southern Thailand. Samples were collected at a depth of 10–15 cm after removing the surface sediment, stored in sterile polyethylene bags, and transported on ice. Ten grams of sediment was placed into a sterile flask and diluted with 90 mL of a sterile 0.85% NaCl solution (RCI Labscan Ltd., Bangkok, Thailand). To facilitate the selection of spore-forming bacteria, the sediment suspension was agitated at 150 rpm for 30 min and then heat-treated at 60 °C for 30 min. The treated mixture was serially diluted up to 10^−6^, and 100 µL aliquots of each dilution were spread-plated onto MH agar (Titan Biotech Ltd., Rajasthan, India). Following a 7-day incubation at 30 °C to allow for colony development, distinct colonies were subcultured to establish pure isolates [52]. A single pure colony was inoculated into MH broth and pre-cultured at 30 °C for 18 h before adjusting the bacterial suspension to an optical density (Genesys 20, Thermo Scientific, Waltham, MA, USA) of 0.1 at 625 nm (OD_625_) using 0.85% NaCl. This prepared suspension was then inoculated into fresh MH broth and incubated with shaking at 150 rpm and 30 °C for 24 h. Subsequently, the culture supernatant was collected by centrifugation at 15,000× *g* and 4 °C for 15 min. The fresh cell-free supernatant was passed through a 0.22 µm cellulose acetate syringe filter (Sigma-Aldrich, Warren, MI, USA) prior to evaluating its antibacterial activity. Antibacterial screening was conducted via an agar well diffusion assay against a panel of bacterial pathogens, including *S. aureus* TISTR 517, *E. coli* TISTR 887, *K. pneumoniae* TISTR 1383, *P. aeruginosa* TISTR 357 (Thailand Institute of Scientific and Technological Research, TISTR, Pathum Thani, Thailand), and three MRSA strains (142, 1096, and 2468). To initiate the assay, suspensions of the indicator pathogens were adjusted to a turbidity of 0.1 OD_625_ (approximately 1 × 10^8^ CFU/mL) and spread evenly onto MH agar plates. Cylindrical wells were created using a sterile cork borer before dispensing 100 µL of the filtered culture supernatant into each well. The sample-loaded plates were left at room temperature to allow for complete absorption of the liquid before being incubated at 37 °C for 24 h. All experiments were performed in triplicate, and antibacterial efficacy was determined by measuring the inhibition zone diameters.

### 4.2. Production Kinetic Studies of Antimicrobial Compounds of the Isolate

Among the isolates tested, only the FNL62 isolate demonstrated antibacterial activity against *S. aureus* and MRSA. To evaluate its production kinetics, an FNL62 preculture (initial OD_625_ of 0.1) was inoculated into 50 mL of MH broth at a 2% (*v*/*v*) ratio. The culture was maintained at 30 °C under constant agitation (150 rpm) for a 7-day period. Cell-free supernatants (CFSs) were collected every 4 h throughout the first 24 h, and subsequently on a daily basis up to day 7. Bacterial growth was monitored by measuring the absorbance at 625 nm. To ensure measurement accuracy, any culture aliquots exhibiting an absorbance approaching or exceeding 0.8 were proportionally diluted in sterile MH broth prior to measurement, and the final OD_625_ values were calculated by multiplying the recorded absorbance by the corresponding dilution factor. Concurrently, the inhibitory potential of each CFS fraction was determined by agar well diffusion assays against *S. aureus* TISTR 517 and three MRSA strains. All assays were conducted in triplicate. Statistical differences in antimicrobial activity across multiple incubation time points were analyzed using a one-way analysis of variance (ANOVA) followed by a post hoc multiple comparisons test (*p* < 0.05). Data are presented as the mean ± SD to reflect the kinetic profile of compound production [53].

### 4.3. Purification of the Mangrove Bacterium-Derived AMP and Amino Acid Sequencing

The orthogonal purification of the AMP from the crude CFS of the FNL62 isolate was conducted using bioassay-guided fractionation. First, proteins from 1 L of crude CFS were precipitated using stepwise ammonium sulfate saturation (25%, 50%, and 75%). The resulting fractions were evaluated for antibacterial activity against MRSA strain 2468 via an agar well diffusion assay. The precipitate fraction exhibiting the highest activity was dissolved and dialyzed against a cation-exchange buffer (50 mM ammonium acetate, 50 mM NaCl, pH 5.0) using 3.5 kDa molecular weight cutoff dialysis tubing (SnakeSkin, Pierce, Rockford, IL, USA) to remove excess salts. Partial purification was then performed using an ÄKTA protein purification system (GE Healthcare Bio-Sciences AB, Uppsala, Sweden). A 2 mL aliquot of the dialyzed sample was loaded onto a 5 mL HiTrap SP column (GE Healthcare Bio-Sciences AB) pre-equilibrated with the cation-exchange buffer. Proteins were eluted isocratically over a 60 mL volume at a flow rate of 1 mL/min, with elution monitored by UV absorbance at 214 nm. The collected fractions were assayed for antibacterial activity, and the active peaks were pooled. For the final purification step via RPC (Ultimate 3000 HPLC, Thermo Fisher Scientific, Waltham, MA, USA), the pooled active cation-exchange chromatography fraction was dialyzed against 0.1% trifluoroacetic acid (TFA) in deionized water and loaded onto an Inertsil ODS-3 C18 column (4.6 × 250 mm; GL Sciences, Tokyo, Japan). The mobile phases consisted of 0.1% TFA in deionized water (Mobile phase A) and 90% acetonitrile containing 0.1% TFA (Mobile phase B). Following the injection of 1 mL of the desalted sample, peptides were eluted using a linear gradient of 0–100% Mobile phase B over a 75 mL volume, with UV detection at 214 nm. Peak fractions were collected, and the solvent was removed by evaporation and lyophilization. The dried compounds were weighed and re-evaluated to confirm antibacterial activity and subsequently stored at −80 °C for further structural and functional characterization, including amino acid sequencing.

De novo amino acid sequencing was employed to elucidate the primary structures of the purified peptides. The active fraction obtained from RPC was subjected to LC-MS/MS analysis using an UltiMate 3000 liquid chromatography system coupled to a high-resolution mass spectrometer (Thermo Fisher Scientific, Waltham, MA, USA). Chromatographic separation was achieved on a reversed-phase C18 Hypersil Gold UHPLC column (4.6 × 30 mm; Thermo Fisher Scientific). The peptides were eluted using a linear gradient of 0–100% acetonitrile containing 0.1% formic acid over 40 min at a flow rate of 300 µL/min. Mass spectra were acquired in both positive and negative ionization modes, with the spray voltage set to 3.2 kV and the capillary temperature maintained at 300 °C. Full MS scans were recorded across an *m*/*z* range of 600–5000. To ensure optimal fragmentation across varying peptide lengths, a stepped normalized collision energy (NCE) was applied. Following data acquisition, de novo peptide sequencing was conducted using PEAKS Studio X software (version 13) (Bioinformatics Solutions Inc., Waterloo, ON, Canada) [54]. The physicochemical properties of the resulting sequences were predicted using the Expasy ProtParam tool [55]. Finally, sequence homology and novelty were evaluated by searching the derived amino acid sequences against established AMP databases, specifically APD6, DRAMP, and DBAASP [56,57,58].

### 4.4. Computational Analysis of FNL62-AMP Secondary Structure

A 3D model of FNL62-AMP was constructed for atomistic modeling using BIOVIA Discovery Studio Visualizer (version 21.1.0.20298) [59]. The peptide structure information was parameterized using the CHARMM36 all-atom force field prior to solvating the single peptide molecule in the 40 × 40 × 40 Å simulation box filled with water molecules (TIP3P water model). System neutralization and the addition of NaCl to a final concentration of 0.15 M were performed using the Solvate and Autoionize extensions in Visual Molecular Dynamics (VMD) [60]. The simulation system was initiated with energy minimization prior to running the simulation in the NPT ensemble at 310 K and 1 atm, utilizing a Berendsen thermostat and barostat with a time step of 2 fs. The secondary structure of the peptide was monitored throughout the trajectory and calculated using the STRIDE algorithm [61].

### 4.5. Determination of Minimum Inhibitory Concentration (MIC) and Minimum Bactericidal Concentration (MBC) of FNL62-AMP

The MIC and MBC of FNL62-AMP were evaluated against *S. aureus* TISTR 517 and three MRSA strains (142, 1096, and 2468) using the broth microdilution method, following Clinical and Laboratory Standards Institute (CLSI) guidelines [62]. Bacterial strains were initially cultured on MH agar at 37 °C for 18 h. A single colony from each culture was suspended in a sterile 0.85% NaCl solution and adjusted to an OD_625_ of 0.1 (approximately 1 × 10^8^ CFU/mL). This suspension was subsequently diluted in cation-adjusted MH broth (CAMHB) to yield a working inoculum of 5 × 10^6^ CFU/mL. Serial dilutions of the purified peptide were also prepared in CAMHB. For MIC determination, a 10 µL aliquot of the working inoculum was added to each well of a 96-well microtiter plate containing the AMP at final concentrations ranging from 0.16 to 8 µg/mL, achieving a total volume of 100 µL per well. Vancomycin and cefoxitin served as reference antibiotic controls. Wells containing the bacterial inoculum without the AMP were included as growth controls, while uninoculated broth served as a sterility control. Following a 24 h incubation at 37 °C, the MIC was defined as the lowest AMP concentration that completely inhibited visible bacterial growth. To determine the MBC, 100 µL aliquots from all wells exhibiting no visible growth were spread-plated onto MH agar and incubated at 37 °C for 24 h. The MBC was defined as the lowest AMP concentration that resulted in a ≥99.9% reduction in the initial bacterial inoculum, indicated by the absence of visible colonies. To prevent false-positive bactericidal results due to peptide carryover, a subculture recovery method was employed. The surface of agar plates exhibiting no colony growth was thoroughly swabbed with a sterile loop and inoculated into 10 mL of fresh Mueller–Hinton (MH) broth. All experiments were performed in triplicate, and the results were reported as the consensus MIC value (mode).

### 4.6. Time-Kill Kinetics of FNL62-AMP

The bactericidal dynamics of FNL62-AMP were assessed using *S. aureus* TISTR 517 and MRSA strains (142, 1096, and 2468). Following standard microdilution procedures, bacterial suspensions were standardized to an initial density of 5 × 10^5^ CFU/mL in CAMHB [62]. These cultures were then exposed to FNL62-AMP at 1×, 2×, 4×, and 8× MIC within 96-well microtiter plates, with peptide-free CAMHB utilized as the untreated control. During continuous incubation at 37 °C, samples were collected at specific intervals over a 24 h period, and the full volume of designated wells was spread directly onto MH agar. After incubating the plates for 24 h at 37 °C, viable colonies were counted. The resulting time-kill profiles were generated by plotting the log_10_ of viable cells (CFU/mL) against the incubation time. To preclude false-positive results, plates exhibiting no visible colony growth were subjected to the subculture recovery procedure described for the MBC determination to definitively verify the bactericidal effect. All assays were conducted in triplicate. Statistical significance between the treated groups and the untreated control across multiple time points was evaluated using a two-way ANOVA followed by Tukey’s post hoc test (*p* < 0.05).

### 4.7. SYTOX Green Membrane Permeabilization Assay

Membrane permeabilization induced by FNL62-AMP was monitored by quantifying the intracellular accumulation of SYTOX Green [63]. Bacterial suspensions grown overnight (16 h) were centrifuged, washed three times in 0.2% CAMHB-supplemented PBS (pH 7.4), and standardized to an OD_625_ of 0.1 (approximately 1 × 10^8^ CFU/mL). Prior to peptide exposure, 100-μL aliquots of the standardized culture were transferred to a 96-well microtiter plate and equilibrated in the dark for 15 min with 10 μM SYTOX Green (Thermo Fisher Scientific, Waltham, MA, USA). FNL62-AMP was subsequently introduced at final concentrations of 0.5×, 1×, 2×, 4×, and 8× MIC. Dye binding to intracellular nucleic acids was recorded continuously over 24 h using a microplate reader configured for an excitation wavelength of 504 nm and an emission wavelength of 523 nm. To establish a baseline for maximum membrane permeabilization, 1% (*v*/*v*) Triton X-100 was included as a positive lysis control. Additionally, a cell-free control containing only FNL62-AMP and SYTOX Green in buffer was monitored in parallel to rule out false-positive fluorescence arising from direct peptide-fluorophore aggregation. Significant variations (*p* < 0.05) relative to the untreated control were determined using a two-way ANOVA followed by Tukey’s post hoc test.

### 4.8. Scanning Electron Microscopy (SEM) Evaluation of FNL62-Treated Bacteria

Morphological alterations induced by FNL62-AMP were evaluated via SEM [8]. Briefly, cultures of *S. aureus* TISTR 517 and MRSA strain 2468 were grown in MH broth for 18 h prior to collection via centrifugation. To eliminate extracellular matrix interference, the recovered cell pellets underwent three consecutive washes in sterile 0.85% NaCl solution. Bacterial suspensions were then prepared in CAMHB and standardized to an OD_625_ of 0.1. Following a subsequent dilution in CAMHB to yield 5 × 10^5^ CFU/mL, the cells were incubated with FNL62-AMP at a concentration equivalent to 1× MIC for 16 h. A parallel treatment involving 1× MIC vancomycin served as the positive control. These antimicrobial agents were prepared and diluted with CAMHB to prevent the medium dilution effect. For microscopic observation, the treated cells were fixed using 2.5% *v*/*v* glutaraldehyde prepared in 0.1 M phosphate buffer (pH 7.2) for 24 h. The fixed samples were subsequently dehydrated through a graded ethanol series, followed by critical point drying (Quorum Technologies Ltd., Lewes, UK) to ensure complete desiccation. Finally, the dried specimens were sputter-coated with gold prior to observation under a scanning electron microscope (Carl Zeiss, Oberkochen, Germany) at 20,000× magnification. To ensure the selected micrographs were representative of the overall morphological changes, the SEM analysis was conducted in three independent biological replicates, with three distinct microscopic fields randomly examined and documented per replicate for each treatment condition.

### 4.9. Stability Assessment of FNL62-AMP

The stability of FNL62-AMP was evaluated across a range of temperatures, pH levels, proteolytic enzymes, and surfactants over incubation periods of 1, 6, and 12 h [54]. Peptide solutions were prepared in sterile purified water at a final concentration of 64 µg/mL. To assess thermal stability, the AMP was incubated at temperatures of 40, 60, 80, and 100 °C, and separately subjected to autoclaving (121 °C, 15 psi) for 15, 30, and 60 min. Enzymatic resistance was determined by treating the peptide with proteinase K, trypsin, or α-chymotrypsin (1 mg/mL, prepared in 50 mM Tris HCl buffer, pH 8.0; Sigma-Aldrich, Warren, MI, USA). For surfactant compatibility, the peptide was exposed to 1% solutions of SDS, CTAB, or Triton X-100 (AppliChem GmbH, Darmstadt, Germany). Parallel control assays containing only the 1% surfactant solutions (without the peptide) were also performed to evaluate the intrinsic antibacterial activity of the surfactants themselves. To evaluate pH stability, the solutions were adjusted to target pH values ranging from 1 to 14 using HCl and NaOH. Following the designated incubation periods, the solutions were neutralized to their original pH (approximately 8). The total volume of all samples was strictly controlled during these adjustments to ensure a unified final peptide concentration of 64 µg/mL across all tested conditions prior to the assay. For all stability assessments, the results are expressed as the mean percentage of residual activity (±SD) relative to the untreated FNL62-AMP control (defined as 100% activity). To allow for direct comparison in the surfactant compatibility assay, the intrinsic activity of the surfactant-only solutions was calculated and expressed as a percentage relative to this same untreated peptide control. Statistical significance was determined using a two-way ANOVA followed by Tukey’s post hoc test (*p* < 0.05).

### 4.10. Phenotypic Characterization and Whole-Genome Sequencing

For phenotypic evaluation, the FNL62 isolate was incubated on MH agar for 12 and 72 h to observe the development of vegetative cells and spores, respectively. Cellular morphology and sporulation capacity were subsequently confirmed through Gram and malachite green staining [64,65]. After genomic DNA extraction, whole-genome sequencing of the isolate was conducted by U2Bio Co., Ltd. (Seoul, Republic of Korea) on an Illumina HiSeq platform (Illumina, San Diego, CA, USA), generating 150 bp paired-end reads. All subsequent bioinformatics workflows were executed on the Galaxy Australia server (version 23.1) [66]. Sequence quality was assessed before and after trimming using FastQC (version 0.12.1) [67], while adapter removal and the filtering of low-quality reads (<30 bp) were performed with Fastp (version 0.23.4) [68]. De novo genome assembly was carried out via the Shovill pipeline (version 1.1.0) using SPAdes (version 3.15.5) [69]. Finally, assembly quality and overall genome completeness were validated with QUAST (version 5.2.0) [70] and CheckM (version 1.0.18) [71]. Taxonomic assignment was performed using a dual-method approach. First, genomic relatedness was evaluated against the TYGS database using the Genome BLAST Distance Phylogeny (GBDP) algorithm [72], allowing for the construction of a phylogenetic tree alongside relevant reference strains to determine evolutionary proximity. To confirm species-level identification, the average nucleotide identity (ANI) between the FNL62 genome and its closest phylogenetic neighbors was calculated using FastANI (version 1.1.0) [73]. This sequence homology was subsequently visualized using the Proksee platform (https://proksee.ca, accessed on 20 January 2026). Functional annotation and the identification of coding sequences (CDSs) were performed using the NCBI Prokaryotic Genome Annotation Pipeline (PGAP) version 6.11 [74]. To evaluate CDS homology between the FNL62 genome and its reference genome, a circular synteny map was generated. This map was plotted based on the Jaccard similarity of the annotated CDSs between the FNL62 genome and its closest taxonomic relative from the TYGS database [75], facilitating the inference of genomic functions and cellular machinery conservation. Furthermore, the best-matched CDS pairs were plotted in a bar graph according to their percentage similarity, visualizing the frequency of conserved functional genes between the two genomes. To elucidate the genetic potential for producing FNL62-AMP and other antimicrobial secondary metabolites, the annotated genome was mined for biosynthetic gene clusters (BGCs) using antiSMASH (version 8.0.4) [76]. The identified BGC profiles were then cross-referenced against established sequences in the GenBank [77] and MIBiG (version 4.0) [78] databases. Finally, a comprehensive circular map illustrating the genomic architecture and annotated features of FNL62 was generated via CGViewBuilder (version 2.0) on the Proksee platform (https://proksee.ca, accessed on 20 January 2026), with relevant genetic elements visualized in color-coded concentric tracks [79].

### 4.11. Molecular Dynamics Simulations of Peptide–Membrane Interactions

For computational modeling and system preparation, an initial atomistic three-dimensional structure of the FNL62-AMP was generated utilizing BIOVIA Discovery Studio Visualizer version 2024 [59]. This single FNL62-AMP molecule was constructed and placed in a 40 × 40 × 40 Å periodic boundary box, solvated with TIP3P water molecules, and supplemented with 0.15 M NaCl. Energy minimization and a production simulation were performed until the peptide reached a stable RMSD, establishing the final hydrated peptide model. To represent the *S. aureus* membrane, a simulated lipid bilayer was generated using the CHARMM-GUI Membrane Builder, comprising a total of 200 lipid molecules distributed symmetrically (100 lipid molecules per leaflet). The specific composition consisted of 80 molecules of 1,2-O-dipalmitoyl-sn-glycero-3-phospho-(1′-rac-glycerol) (DPPG) (40%), 104 molecules of 1,2-O-dipalmitoyl-sn-glycero-3-phospho-rac-(3-lysyl(1′-glycerol)) (Lysyl-DPPG) (52%), and 16 molecules of 1,1′,2,2′-tetramyristoyl cardiolipin (TMCL) (8%). Prior to simulating the peptide–membrane interactions, this membrane system was independently equilibrated for 1000 ns until a steady RMSD was achieved, confirming full structural equilibration. The hydrated peptide and membrane system assembly was executed via Packmol (version 21.0.1) [80]. Initial atomistic models of both the peptide and the membrane were parameterized utilizing the CHARMM36 all-atom force field [60] prior to transformation into a coarse-grained model and parameterization using the MARTINI force field [81]. To investigate single-peptide interactions, one FNL62-AMP molecule was positioned 30 Å above the extracellular leaflet of the constructed *S. aureus* membrane along the *z*-axis (50 Å from the center of the membrane) and solvated with 9810 water molecules. Molecular dynamics simulations were conducted under the isothermal-isobaric (NPT) ensemble at 310 K and 1 atm. Temperature and pressure were maintained utilizing a Berendsen thermostat and barostat, respectively, with an integration time step of 20 fs. The system was energy-minimized prior to executing the production run for the single-peptide membrane system. For trajectory analysis and the extraction of biophysical metrics, processing and quantitative evaluations were executed using custom Python scripts (version 3.9.0) and the MDAnalysis library (version 2.1) [82]. To evaluate the structural stability and convergence of the simulated systems, the RMSD of both the lipid bilayer and the FNL62-AMP assemblies was computed across the simulation timelines. The diffusive behavior and translational mobility of the peptide and the lipid matrix were monitored via MSD analysis, while peptide penetration into the membrane was monitored by tracking the distance between the COM of the peptide and the membrane along the *z*-axis [83]. Specific peptide–membrane interactions were determined by calculating the contact frequency between individual amino acid residues and the lipid bilayer using a distance cutoff of 6 Å, identifying the key residues mediating adsorption [84].

For the exploration of FNL62-AMP concentration-dependent behavior and membrane interactions, an aqueous self-assembly system was prepared. A coarse-grained model comprising 64 FNL62-AMP molecules was randomly dispersed in a 100 × 100 × 100 Å cubic simulation box, solvated with the MARTINI water model, and adjusted to 0.15 M NaCl [81]. The system was subsequently simulated following the single-peptide aqueous protocol. The self-aggregation kinetics of the peptide in bulk solvent (5357 water molecules) were simulated and the aggregation phenomenon was monitored by quantifying the SASA over time. Furthermore, the *R_g_* of the 64-peptide aggregate was calculated to confirm its consolidation into a compact micellar architecture [16,85]. The system was subsequently simulated following the single-peptide aqueous protocol. To evaluate self-aggregation kinetics in the bulk solvent, this initial phase was simulated until structural equilibrium was achieved. The progression of the aggregation phenomenon was monitored by quantifying the solvent-accessible surface area (SASA) and the radius of gyration (*R_g_*) over time. The criterion for equilibrium was defined as the point at which the SASA and *R_g_* values plateaued into a steady baseline. The resulting peptide macro-aggregate model was then introduced to the *S. aureus* membrane model to investigate interaction phenomena. Specifically, the equilibrated 64-peptide aggregate generated during the aqueous simulation was positioned 40 Å above the lipid bilayer, which was solvated with 12,888 water molecules. All coarse-grained simulation systems were subsequently set up according to the established protocol for single peptide–membrane interactions [86,87]. To evaluate the topological distortions induced by FNL62-AMP insertion, the spatial z-coordinates of the phospholipid headgroups were mapped onto a two-dimensional grid using Python scripts utilizing the MDAnalysis and SciPy libraries. Specifically, the spatial coordinates (x, y, z) of the upper leaflet phosphate headgroups relative to the membrane’s center of mass were extracted and projected onto a uniform two-dimensional spatial grid. To generate a continuous surface topography, the discrete atomic coordinates were processed using linear interpolation. The interpolated Z-coordinates were subsequently normalized against the average baseline height of the unperturbed membrane. This specific topological mapping quantified localized deviations in membrane depth in Ångströms (Å), allowing the resulting surface profiles to accurately capture the upward displacement of the lipid matrix and the downward dimpling associated with peptide translocation and subsequent membrane rupture [40,88]. All simulation trajectories were visualized and rendered using Visual Molecular Dynamics (VMD) software (version 1.9.4) [89].

### 4.12. Preliminary In Vitro Characterization of Peptide Aggregation

Dynamic light scattering (DLS) was used to evaluate the aggregate size of the self-assembled peptide structures in solution [46]. Experiments were conducted using a Zetasizer Nano ZS (Malvern Instruments, Worcestershire, UK). FNL62-AMP samples were prepared by ultra purified water at varying concentrations corresponding to 1×, 2×, 4×, and 8× MIC. DLS measurements were performed at 25 °C utilizing a 173° backscatter angle following a 120 s equilibration time. The intensity-based z-averaged hydrodynamic diameters were reported based on 11 consecutive scans per measurement. All sample concentrations were measured independently in triplicate to ensure statistical reproducibility. Fluorescence spectroscopy was performed to monitor the concentration-dependent aggregation and the formation of localized peptide microenvironments; intrinsic steady-state fluorescence was measured using an FP-8200 spectrofluorometer (Jasco Ltd., Heckmondwike, UK) [90,91]. The intrinsic fluorescence intensity of the peptide aggregates at concentrations of 1×, 2×, 4×, and 8× MIC was recorded using an excitation wavelength of 410 nm and an emission wavelength of 500 nm. All spectroscopic measurements were performed using excitation and emission slits with a nominal band pass of 1 nm. The background fluorescence of the buffer was subtracted, and all measurements were conducted in triplicate.

## 5. Conclusions

Marine ecosystems, particularly dynamic mangrove environments, represent a highly valuable and largely untapped reservoir for the discovery of novel bioactive compounds. Highlighting the potential of these unique ecological niches, the genomic identification of a novel *Brevibacillus* species and the characterization of FNL62-AMP offer a promising starting point for antimicrobial discovery. Supported by molecular dynamics simulations, current evidence suggests that the peptide acts via a rapid, physical membrane-lytic pathway, modeled by the formation of a macro-aggregate that follows a carpet-like mechanism at the membrane interface. The aggregated peptide formation is supported by the preliminary results of dynamic light scattering and fluorescence. However, these computational findings provide predictive insights that necessitate further in vitro biophysical validation. Furthermore, while FNL62-AMP exhibits remarkable thermal stability, its inherent tendency to form hydrophobic aggregates and its high sensitivity to specific surfactants pose distinct challenges for standard formulation. Consequently, the mechanistic and interaction profiles elucidated in this study serve as an essential foundational framework. This biophysical knowledge will be crucial for guiding future research toward the development of antimicrobial peptides for clinical application against multidrug-resistant infections.

## Figures and Tables

**Table 1:** Purification balance sheet of the active antimicrobial compound isolated from the FNL62 strain.

Purification Step	Total Volume (mL)	Total Dried Weight (mg)	Arbitrary Activity (AU/mL)	Total Activity (AU)	Specific Activity (AU/mg)	Purification Factor (Folds)	Yield (%)
Cell-free supernatant of bacterial culture (crude supernatant	987.54	964.82	20	19,750.80	20.47	1	100
Precipitation	42.32	137.27	320	13,542.40	98.66	4.82	68.57
Cation exchange chromatography	104.58	39.33	80	8366.40	212.72	10.39	42.36
Reversed-phase chromatography	29.22	9.18	80	2337.60	254.64	12.44	11.84

**Table 2:** MIC and MBC values of FNL62-AMP determined by microdilution assay.

Tested Samples	FNL62-AMP		Vancomycin		Cefoxitin	
	MIC (μg/mL)	MBC (μg/mL)	MIC (μg/mL)	MBC (μg/mL)	MIC (μg/mL)	MBC (μg/mL)
*S. aureus* TISTR 517	0.5	2	2	2	2	2
MRSA strain 142	1	4	2	2	>64	>64
MRSA strain 1096	1	4	2	2	>64	>64
MRSA strain 2468	1	4	2	2	>64	>64

**Table 3:** Residual anti-MRSA activity (%) of FNL62-AMP (64 µg/mL) under diverse thermal, enzymatic, surfactant, and pH conditions over a 12 h period.

Treatment Conditions	Residual Activity (%) of FNL62-AMP (64 µg/mL) Against MRSA Strain 2468 at Various Time Points		
	(Mean ± SD; *n* = 3)		
Effect of temperature variation	1 h	6 h	12 h
40 °C	100.59 ± 1.64	97.29 ± 1.43	100.11 ± 0.20
60 °C	99.05 ± 1.08	98.35 ± 2.65	96.79 ± 1.30
80 °C	96.69 ± 2.85	96.23 ± 2.13	89.79 ± 1.70 * ^‡^
100 °C	93.14 ± 2.02 *	93.99 ± 2.12 *	82.34 ± 2.21 * ^‡^
Non-treatment	100.00 ± 0.54	100.00 ± 0.35	100.00 ± 0.87
Effect of autoclave condition	15 min	30 min	1 h
121 °C and 15 psi	94.69 ± 2.32 *	90.33 ± 1.67 *	86.79 ± 1.67 * ^‡^
Non-treatment	100.00 ± 0.54		
Effect of protease digestion	1 h	6 h	12 h
FNL62-AMP with proteinase K (1 mg/mL)	99.63 ± 1.35	98.62 ± 0.60	92.65 ± 0.75 * ^‡^
FNL62-AMP with trypsin (1 mg/mL)	99.88 ± 0.22	98.97 ± 0.91	98.55 ± 1.66
FNL62-AMP with α-chymotrypsin (1 mg/mL)	95.63 ± 1.56 *	88.76 ± 1.96 * ^†^	79.52 ± 3.36 * ^‡^
Non-treatment	100.00 ± 0.99	100.00 ± 0.40	100.00 ± 0.55
Effect of surfactant	1 h	6 h	12 h
FNL62-AMP with CTAB (1% *w*/*v*)	69.57 ± 1.29 * ^§^	69.24 ± 1.81 * ^§^	64.76 ± 1.84 * ^§^
FNL62-AMP with SDS (1% *w*/*v*)	87.24 ± 0.85 * ^§^	87.87 ± 1.91 * ^§^	86.22 ± 1.48 * ^§^
FNL62-AMP with Triton X-100 (1% *w*/*v*)	101.72 ± 2.37 ^§^	101.35 ± 2.33 ^§^	97.44 ± 0.63 ^§^
CTAB (1% *w*/*v*) alone	79.88 ± 1.89 *	80.64 ± 2.61 *	80.98 ± 2.76 *
SDS (1% *w*/*v*) alone	103.93 ± 2.45	103.06 ± 1.81	103.05 ± 2.01
Triton X-100 (1% *w*/*v*) alone	0.00 ± 0.00 *	0.00 ± 0.00 *	0.00 ± 0.00 *
Non-treatment	100.00 ± 0.56	100.00 ± 0.74	100.00 ± 0.92
Effect of pH variation	1 h	6 h	12 h
1.0	93.39 ± 1.34 *	91.03 ± 1.85 *	87.80 ± 0.34 *
2.0	95.81 ± 2.29 *	93.37 ± 0.64 *	87.12 ± 0.90 * ^‡^
4.0	100.89 ± 0.79	93.73 ± 0.98 * ^†^	89.49 ± 1.48 * ^‡^
6.0	99.75 ± 1.96	97.67 ± 1.74	98.42 ± 2.18
7.4	100.64 ± 1.14	98.40 ± 1.06	99.66 ± 3.23
8.0	99.24 ± 0.58	99.26 ± 1.47	99.89 ± 1.09
10.0	99.87 ± 2.12	97.30 ± 0.93	90.06 ± 1.71 * ^‡^
12.0	99.49 ± 1.66	97.17 ± 1.18	90.17 ± 1.89 * ^‡^
14.0	99.24 ± 1.54	94.10 ± 1.33 *	87.01 ± 2.40 * ^‡^
Non-treatment	100.00 ± 0.44	100.00 ± 0.77	100.00 ± 0.63

Statistical significance (*p* < 0.05, two-way ANOVA with Tukey’s post hoc test) of activity retention was determined for the following comparisons: treatments versus the control within the same time point (*); the 6 h (^†^) and 12 h (^‡^) incubations versus the 1 h time point; and the surfactant alone versus the surfactant with FNL62-AMP mixture (^§^).

## Data Availability

Data are contained within the article.

## Supplementary Materials

The following supporting information can be downloaded at https://www.mdpi.com/article/10.3390/md24090309/s1. Table S1: De novo amino acid sequence, fragment ion assignments, and local confidence scores for FNL62-AMP derived from MS2 spectra; Table S2: Observed m/z values and mass errors (Da) for the b- and y-ion fragmentation series of FNL62-AMP.
