# Supplementary material for: A Marine Brevibacillus-Derived Membrane-Lytic Peptide: Molecular Insight and Biophysical Characterization of a Novel AMP, FNL62-AMP
Source: Mar Drugs. 2026 Sep 4;24(9):309. doi: 10.3390/md24090309 (PMC13608124; doi:10.3390/md24090309)
Supplement: Supplementary file 1 [file marinedrugs-24-00309-s001.zip › marinedrugs-4502936-supplementary.pdf]

A Marine *Brevibacillus*-Derived Membrane-Lytic Peptide: Molecular Insight and Biophysical Characterization of a Novel AMP, FNL62-AMP

Namfa Sermkaew [1,2], Apichart Atipairin [1,2], Sucheewin Krobthong [3], Chanat Aonbangkhen [3,4], Yodying Yingchutrakul [5], Jumpei Uchiyama [6] and Nuttapon Songnaka [1,2,*]

Table S1. *De novo* amino acid sequence, fragment ion assignments, and local confidence scores for FNL62-AMP derived from MS2 spectra.

Position (b)	b	b-H2O	b-NH3	b[2+]	Sequence	y	y-H2O	y-NH3	y[2+]	Position (y)	ALC (%)
1	114.09092				L					6	79
2	227.1752			114.09092	L				331.25165	5	93
3	340.25284	322.24448	323.24915		L	548.41693				4	92
4		435.33286	436.32507	227.1752	L	435.33286				3	92
5					F	322.24448	304.23679			2	52
6					R					1	60

Table S2. Observed m/z values and mass errors (Da) for the b- and y-ion fragmentation series of FNL62-AMP.

Ion Name	Position	Ion m/z	m/z Error
b1	1	114.09092	-0.00042
b2	2	227.1752	-0.0002
b3	3	340.25284	-0.00662
b3-H2O	3	322.24448	-0.00443
b4-H2O	4	435.33286	-0.00009
b3-NH3	3	323.24915	0.01669
b4-NH3	4	436.32507	0.00854
b2[2+]	2	114.09092	0.00322
b4[2+]	4	227.1752	0.00345
y4	4	548.41693	0.06146
y3	3	435.33286	0.06143
y2	2	322.24448	0.05713
y2-H2O	2	304.23679	0.06
y5[2+]	5	331.25165	0.03189
